# Transcriptome and TCR Repertoire Measurements of CXCR3^+^ T Follicular Helper Cells Within HIV-Infected Human Lymph Nodes

**DOI:** 10.3389/fimmu.2022.859070

**Published:** 2022-05-06

**Authors:** Chenfeng He, Michael J. Malone, Ben S. Wendel, Ke-Yue Ma, Daniel Del Alcazar, David B. Weiner, Philip L. De Jager, Perla M. Del Río-Estrada, Yuria Ablanedo-Terrazas, Gustavo Reyes-Terán, Laura F. Su, Ning Jiang

**Affiliations:** ^1^Department of Biomedical Engineering, Cockrell School of Engineering, University of Texas at Austin, Austin, TX, United States; ^2^Department of Bioengineering, University of Pennsylvania, Philadelphia, PA, United States; ^3^Interdisciplinary Life Sciences Graduate Program, University of Texas at Austin, Austin, TX, United States; ^4^McKetta Department of Chemical Engineering, Cockrell School of Engineering, The University of Texas at Austin, Austin, TX, United States; ^5^Department of Medicine, Division of Rheumatology, Perelman School of Medicine, Institute for Immunology, University of Pennsylvania, Philadelphia, PA, United States; ^6^Corporal Michael J Crescenz Veterans Affairs Medical Center, Philadelphia, PA, United States; ^7^Vaccine and Immunotherapy Center, Wistar Institute, Philadelphia, PA, United States; ^8^Columbia University Medical Center, Center for Translational and Computational Neuroimmunology, New York, NY, United States; ^9^Departamento de Investigación en Enfermedades Infecciosas, Instituto Nacional de Enfermedades Respiratorias, Ciudad de México, Mexico; ^10^Comisión Coordinadora de Institutos Nacional de Salud y Hospitales de Alta Especialidad, Secretaría de Salud, Ciudad de México, Mexico; ^11^Institute for Immunology, University of Pennsylvania Perelman School of Medicine, Philadelphia, PA, United States

**Keywords:** CXCR3, follicular-helper T cells (T_FH_), TCR repertoire, RNA-seq, HIV

## Abstract

Follicular-helper T cells (T_FH_) are an essential arm of the adaptive immune system. Although T_FH_ were first discovered through their ability to contribute to antibody affinity maturation through co-stimulatory interactions with B cells, new light has been shed on their ability to remain a complex and functionally plastic cell type. Due to a lack sample availability, however, many studies have been limited to characterizing T_FH_ in mice or non-canonical tissue types, such as peripheral blood. Such constraints have resulted in a limited, and sometimes contradictory, understanding of this fundamental cell type. One subset of T_FH_ receiving attention in chronic infection are CXCR3-expressing T_FH_ cells (CXCR3^+^T_FH_) due to their abnormal accumulation in secondary lymphoid tissues. Their function and clonal relationship with other T_FH_ subsets in lymphoid tissues during infection, however, remains largely unclear. We thus systematically investigated this and other subsets of T_FH_ within untreated HIV-infected human lymph nodes using Mass CyTOF and a combination of RNA and TCR repertoire sequencing. We show an inflation of the CXCR3^+^T_FH_ compartment during HIV infection that correlates with a lower HIV burden. Deeper analysis into this population revealed a functional shift of CXCR3^+^T_FH_ away from germinal center T_FH_ (GC-T_FH_), including the altered expression of several important transcription factors and cytokines. CXCR3^+^T_FH_ also upregulated cell migration transcriptional programs and were clonally related to peripheral T_FH_ populations. In combination, these data suggest that CXCR3^+^T_FH_ have a greater tendency to enter circulation than their CXCR3^-^ counterparts, potentially functioning through distinct modalities that may lead to enhanced defense.

## Introduction

Follicular-helper T cells (T_FH_) provide B cells with the necessary costimulatory signals for their affinity maturation of broadly neutralizing antibodies (BNAbs) (1), a critical component of host defense. Despite high levels of circulating IgG in HIV-infected patients (2, 3), BNAbs and natural viral control are uncommon (4, 5). Given the importance of T_FH_ contribution in a successful antibody response, it is possible their inadequacy during HIV infection leads to a lack of effective and timely B cell help.

Surprisingly, sheer numbers of T_FH_ do not appear to be the problem. In fact, T_FH_ exist at high frequencies during untreated HIV infection (6, 7), suggesting a qualitative, rather than quantitative, impotence. In fact, we and others have shown that T_FH_ in HIV^+^ patients are functionally and phenotypically distinct compared to healthy donors (8, 9), even during effective anti-retroviral therapy (ART). In addition to their altered cell state, HIV-specific T_FH_ appear to be slow-acting, requiring prolonged exposure to antigen likely during a period of uncontrolled viral replication (10). Further complicating matters, T_FH_ are readily infected by HIV. Several studies have implicated their role as an important reservoir for latent infection (11–14) and subsequent viral persistence. This complex interplay between host and virus have made it a great challenge to understand the pitfalls of the immune system during HIV infection.

A high degree of cellular plasticity in the T_FH_ compartment (15–17) has also led to our incomplete understanding of its role in HIV infection. T_FH_ are generally identified by their expression of CXCR5, a chemokine receptor necessary for proper follicular localization in the lymph node (LN) (18, 19). However, several less understood subsets have been characterized based on other cell surface markers. For example, in experimental autoimmune encephalomyelitis (EAE), a mouse disease model for multiple sclerosis, IL17a-expressing T_FH_ have been found to infiltrate the central nervous system and exacerbate inflammation and disease (20). In contrast, antigen-specific CCR6-expressing T_FH_ were observed at high levels in SARS-COV-2 infection, correlating with less severe disease (21). Interestingly, CCR6^+^T_FH_ in this context, normally described as TH17-like, seldom expressed IL17a. In an opposite functional context, regulatory T_FH_ have also been described as FOXP3^+^CXCR5^+^T_FH_ that appear to suppress the germinal center reaction (22). Recently, we described an activated CXCR5^-^ T_FH_-like population in LNs that accumulates in HIV infection. These cells specifically co-express high levels of ICOS and PD-1, are clonally related to T_FH_ and can promote the production of HIV-specific antibodies from B cells *in vitro* (9). We speculate that these cells, having a heightened migratory gene program, may infiltrate inflamed LNs and modify the B cell response; however, our understanding of their role in infection and relationship with other CXCR5^+^ T_FH_ populations remains insufficient.

Another T_FH_ subset that has received a fair amount of attention expresses the bona fide T_H1_ marker, CXCR3. A recent study depicting the clonal relationships between T_FH_ subsets in the tonsils and blood of healthy humans found a strong clonal connection between CXCR3-expressing T_FH_ (CXCR3^+^T_FH_) and circulating T_FH_ (cT_FH_) (23). In agreement with previous publications (24, 25), the authors posit that the origin of germinal center T_FH_ (GC-T_FH_) may be CXCR3^+^T_FH_ that initially encountered antigen in the periphery. Interestingly, other studies have shown CXCR3^+^T_FH_ to be enriched during chronic SIV infection in non-human primates (26, 27), in the blood during HIV infection (28, 29), and after influenza vaccination (30). Given their inflation in non-steady state conditions, it is possible that studying CXCR3^+^T_FH_ in these contexts may reveal a clearer picture of their role leading up to and during their B cell interactions. Furthermore, with an altered cell state appearing to be a hallmark of T_FH_ in HIV disease progression (8), it is possible that understanding these alternative T_FH_ subsets will bring light to novel mechanisms of disease and/or host defense. A systematic study of CXCR3^+^T_FH_ during perturbation, across multiple tissues in humans is thus of critical need to fully understand their function within the adaptive immune system.

Although blood studies in humans and work done with model organisms have laid the foundation for our understanding of T_FH_, their results have been contradictory at times. For example, Velu et al. observed similar helper function between LN CXCR3^+^T_FH_ and CXCR3^-^T_FH_ to induce SIV-specific IgG *in vitro* (27), while other studies suggested that CXCR3^+^T_FH_ from human blood provide poor B cell help (31, 32). It is likely that context, such as tissue type and disease state, has a great impact on the role and function of T_FH_. As such, our grasp remains limited without clear delineation of the relationship between cT_FH_ and their native, lymphoid-resident counterparts in both health and disease. In this study, we aim at narrowing this deficit in knowledge using a combination of Mass CyTOF and TCR repertoire and RNA sequencing on several different T cell populations from paired LNs and peripheral blood samples in untreated HIV^+^ patients. Importantly, we find that CXCR3^+^T_FH_ are both inflated, phenotypically distinct, and correlate with lower HIV burden. We thus emphasize our analysis on this population and its relationship to several better characterized T cell populations within the human LN. Our multi-parameter approach revealed enhanced proliferative potential, an upregulation of cell migration pathways, and strong cross-tissue and cross-phenotype clonal relationships within CXCR3^+^T_FH_. These data suggest the potential of CXCR3^+^T_FH_ to expand within the LN, enter circulation and possibly contribute to host defense through alternative processes than their canonical GC-T_FH_ counterparts. Further investigation into the impact of CXCR3^+^T_FH_ on viral load and spread over time with a larger, longitudinally tracked cohort will be important to unravel specific mechanisms that these may operate through.

## Materials and Methods

### Study Subjects

Subjects included in this study are from a subset of patients recruited for our previous study (8). Briefly, LN samples from HIV^+^ donors were excised from palpable cervical LNs for clinical diagnostic workup in Mexico. HD samples were de-identified mesenteric or inguinal lymph nodes from the Cooperative Human Tissue Network (CHTN). Twenty-two HIV^+^ donor LNs and nine HD LNs were used for Mass CyTOF experiments. Considering sample availability and size, LN and PBMC samples from an independent, but semi-overlapping cohort (**Tables S1, S5**) were collected from LN and PBMC samples were collected from seven non-ART and one ART-treated HIV^+^ patients. Sample sizes were not pre-specified but dictated by the availability of the sample. All samples from HIV+ patients were de-identified and were obtained in accordance with the Declaration of Helsinki after obtaining written informed consent of participants and as part of protocol B03-16, which was reviewed and approved by the Research Committee and the Ethics in Research Committee of the National Institute of Respiratory Diseases “Ismael Cosío Villegas”, Mexico City.

### Mass CyTOF and Analysis

**Figure 1:**
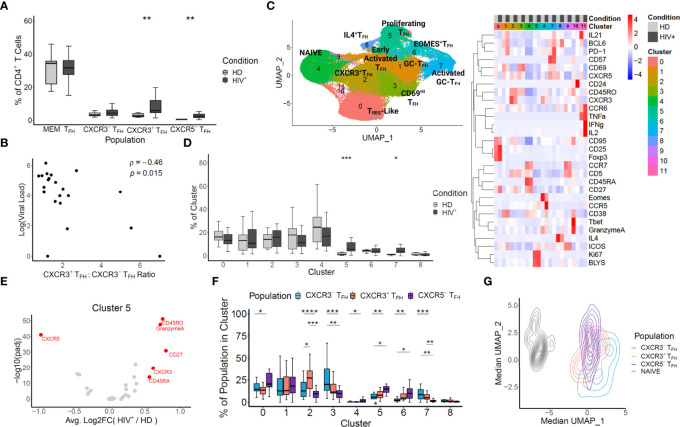
Mass CyTOF reveals an enrichment of non-canonical CXCR3-expressing TFH unique to HIV^+^ lymph nodes. **(A)** T_FH_ populations were *in silico* sorted based on the gating illustrated in **Supplementary Figure 1**. The frequencies of HIV^+^ and HD LN cells within each population were compared with a t-test. **(B)** CXCR3^+^T_FH_, CXCR3^-^T_FH_, CXCR5^-^T_FH_ and T cells were *in silico* sorted, visualized with UMAP, and clustered (left). Clusters were annotated based on the heatmap of the markers shared across each experiment (right). **(C)** Log pVL and the ratio of CXCR3^+^T_FH_ to CXCR3^-^T_FH_ frequencies were compared for each donor using a Spearman’s rank correlation coefficient. **(D)** The frequencies of HIV^+^ patient cells versus HD cells for each donor within each cluster from B were compared using a Wilcox test. **(E)** Differential expression HIV^+^ versus HD derived cells in cluster 5 was calculated for each marker based on a likelihood-ratio test. **(F)** Pairwise frequencies of *in silico* sorted T_FH_ within each cluster (HIV^+^ donors only) was compared using a t-test. **(G)** The median UMAP coordinates were calculated for each donor *in silico* sorted populations and projected onto the same coordinates as in **(B)** Significance: ****p < 0.0001; ***p < 0.001; **p < 0.01; *p < 0.05; ns p ≥ 0.05.

Mass CyTOF data were collected as described in a previous publication (8), except that the cells were not stimulated prior to staining. Data were collected and normalized as described there. In analysis specific to this paper, cells were subset by drilling down on the gates defined in **Figure S1** with FlowJo™ v10.8.0 Software (BD Life Sciences) and exported as individual FCS files. FCS files were then read into R using the “flowCore” package and analyzed using custom scripts. Dimensional reduction and clustering were done using the R package “Seurat”. Cluster annotations and rational are described in **Table S2**. Boxplots and UMAP plots were generating using the R package “ggplot2”. The heatmap in **Figure 1** was made with the R package “pheatmap” (33). Statistical analyses were done using the R package “rstatix”. Final plots were compiled in Biorender.

### Cell Staining and Sorting by Flow Cytometry

Cell staining and sorting were performed as previously described (9). Briefly, cryopreserved LN cell suspensions were freshly thawed. A BD FACSARIA was used to sort live CD4^+^ T cells as CD3^+^CD4^+^CD8^-^CD14^-^CD19^-^. Then, from the live CD4^+^ T cell population, naïve (CD45RO^-^CXCR5^-^CD57^-^CCR7^+^), CXCR3^+^T_FH_ (CD45RO^+^CXCR5^+^CCR7^-^PD1^+^CXCR3^+^), CXCR3^-^T_FH_ (CD45RO^+^CXCR5^+^CCR7^-^PD1^+^CXCR3^-^), GC-T_FH_ (CD45RO^+^CXCR5^+^CD57^+^) and CXCR5^-^T_FH_ (CD45RO^+^CXCR5^-^PD1^+^ICOS^+^) populations were sorted directly into lysis buffer and stored at -80°C until further use.

### RNA Purification

Total RNA was purified using AllPrep DNA/RNA Mini and RNeasy MiniElute kits (Qiagen) according to the manufacturer’s instructions. Purified RNA quality was evaluated using Agilent RNA 6000 Pico Kit. Samples were then either supplemented with RNase inhibitor (RNase OUT, Thermofisher Scientific) and stored at -80°C or taken directly to reverse transcription.

### Bulk TCRβ Sequencing and Analysis

TCRβ repertoire sequencing libraries were prepared and sequenced as described previously (8). Briefly, 30% of purified RNA was used for reverse transcription. Molecular identifiers (MIDs) were added to cDNA templates during reverse transcription using a TRBC-binding primer with 12 random nucleotides and a partial Illumina adapter (**Table S4**). PCR1 was carried out using multiplexed TRBV-binding primers. A second round of PCR was then used to append final Illumina sequencing adapters to the TCRβ junction-containing inserts. Final libraries were sequenced on an Illumina paired end 150x150 configuration at a minimum depth of 10 reads/cell. After sequencing, reads were clustered based on their MID and TCRβ sequence similarity. Consensus sequences were then built to correct PCR and sequencing errors as described (34). TCRβ sequencing information is summarized in **Table S5**. The CDR3 blast module MIGEC (35) was used for TRBV/J and CDR3 annotation. Bhattacharyya coefficient (36) was used to measure TCR repertoire similarity. Circos plots were generated using circlize R package (37).

### RNA Sequencing and Analysis

RNA-Seq libraries were prepared using a protocol modified from SMART-seq2 (SSII) (38). Briefly, 2ul of purified total RNA was reverse transcribed using a poly-T primer fused to the ISPCR handle (**Table S6**; RT_dT30VN). Second-strand synthesis was then done using the SSII template-switching oligo (**Table S6**; SSII_TSO). The following program was used: 42°C RT for 90min, 10 cycles of 50°C for 2min, 42°C for 2min and then 70°C for 15min. cDNA amplification was then done using KAPA HIFI and the ISPCR handle (**Table S6**; RT_TSPCR) with the following program: 98°C initial denature for 3min, 16 cycles of 98°C denature for 20sec, 64°C annealing for 30sec, 72°C extension for 6min and 72°C final extension for 5min. The PCR product was then purified using AmpureXP beads (Agencourt), according to the manufacturer’s protocol. Purified PCR product was then diluted to 0.1 – 0.3 ng/ul and tagmented using the Nextera XT kit (Illumina) with a reduced reaction volume. Briefly, 1.25 ul of diluted sample was used in a 5ul total reaction volume and fragmented for 10min at 55°C, and then held at 10°C. The reaction was then neutralized by adding 1.25 ul of NT buffer. Final libraries were then generated from the tagmentation product using Nextera adapters (**Table S6**) and the following program: 72°C for 3 min, 95°C denature for 3sec, 12 cycles of 95°C denature for 10sec, 55°C annealing for 30sec, 72°C extension for 1min and 72°C final extension for 5min. Indexed libraries were then purified using Ampure XP beads according to manufacturer’s instructions and quantified with an Agilent High Sensitivity DNA kit. The final libraries were pooled and sequenced on Illumina HiSeq (150x150) with a minimum depth of about 200 reads per cell. Each library was split into two technique replicates and sequenced independently to ensure the reliability of RNA sequencing results.

Sequencing reads were aligned to human reference genome GRCh38.p5 using RSEM (39). Differentially expressed genes were analyzed using DESeq2 (40). GSVA, a non-parametric unsupervised method that quantifies the relative enrichment of selected pathways, was performed using the R package, GSVA (41). Gene Set Enrichment Analysis (GSEA) on selected pathways or gene sets were performed with the R package fgsea (42). Tonsil Tfh signatures (GSE50391, CXCR5^high^CD45RO^+^ versus CXCR5^-^ tonsil samples) were created using the GEO2R online tool (https://www.ncbi.nlm.nih.gov/geo/geo2r/). Metascape (43) and NetworkAnalyst (44) was used to determine GO pathway enrichment.

### Quantification of HIV Transcripts From Bulk RNA-Seq

HIV transcripts were quantified from bulk RNA-Seq following the stepwise procedures (**Figure S19**) similar to the method outlined in Chang et al. (45). Briefly, reads that could not be aligned to the human genome were mapped to the HIV genome (NC_001802.1) with the gapped aligner software HISAT2 (46), accounting for splice junction events. Quantification of HIV transcript expression was calculated by normalizing the HIV genome mappable reads to human genome mappable reads and the input cell number. For a more reliable quantification of HIV copies in each library, the correlation between two replicates was first evaluated (**Figure S20**), then the HIV copy numbers of both technical replicates were averaged.

### Statistical Analysis

A paired sample two-tailed Student’s t-test was used for pairwise comparisons, with a P value less than 0.05 as a cutoff to determine statistical significance. These comparisons include CXCR3^+^/CXCR3^-^T_FH_ percentages in **Figure S10C**, TCR repertoire similarities in **Figures 4B, C** and HIV copies in **Figure 5A**. For gene set enrichment analysis, CAMERA (47) (Correlation Adjusted MEan RAnk gene set test) was used to calculate enrichment significance. Pearson correlation was used to determine the degree of association.

## Results

### Mass CyTOF Reveals an Enrichment of Non-Canonical CXCR3-Expressing T_FH_ Unique to HIV^+^ Lymph Nodes

We first set out to determine if, in concordance with SIV infection, CXCR3^+^T_FH_ were inflated in human LN during HIV infection. We used a 29 marker Mass CyTOF panel to survey T cells from 22 HIV^+^ and 9 healthy donor LNs (Characteristics of the donors are listed in **Table S1**). We compared the frequencies of four T_FH_ populations using the gating strategies described in **Figure S1**. Two of these populations, denoted as MEM T_FH_ and CXCR3^-^T_FH_, are defined as CD45RO^+^CXCR5^+^ and CD45RO^+^CXCR5^+^PD1^+^CXCR3^-^ CD4^+^ T cells, respectively. These two gating schemes are frequently used when characterizing T_FH_ (8, 32, 48). The remaining two populations, CXCR3^+^T_FH_ and CXCR5^-^T_FH_, are defined as CD45RO^+^CXCR5^+^PD1^+^CXCR3^+^ and CD45RO^+^CXCR5^-^PD1^+^ICOS^+^ CD4^+^ T cells, respectively. CXCR3^+^T_FH_ have been characterized in rhesus macaques (RMs) and human blood (27, 31, 32), while CXCR5^-^T_FH_ were recently described as functional T_FH_ in human LN and blood during HIV infection (9). Amongst the four populations analyzed, CXCR3^+^T_FH_ and CXCR5^-^T_FH_ were significantly enriched in HIV^+^ patient LNs versus healthy donor LNs (**Figure 1A**). It should be noted, however, that all LNs from HIV^+^ donors were cervical, whereas HD LNs were derived from a mix of distinct anatomical locations (**Table S1**). Regardless, given their high frequency over steady state, we suspected these two T_FH_ subsets may play a role in HIV infection. To directly address this question, we compared the frequencies of T_FH_ populations with each patient’s plasma viral load (pVL) (**Figure S2**). Interestingly, the only metric correlated to pVL is the ratio of CXCR3^+^T_FH_ to CXCR3^-^T_FH_ (**Figure 1B**), suggesting that donors with greater CXCR3^+^T_FH_ populations may be better at controlling virus.

To gain insight into the role and interplay of these T_FH_ populations in HIV infection, we sought to delve deeper into the Mass CyTOF data. To do so, we subset the data by drilling down on four T cell populations (CXCR3^+^T_FH_, CXCR3^-^T_FH_, CXCR5^-^T_FH_ and Naïve) described in **Figure S1**, effectively sorting these populations *in silico*. We then projected the *in silico* sorted populations onto two dimensions using Uniform Manifold and Projection (UMAP). At a first glance, cells derived from HIV^+^ donors occupied several distinct locations on the UMAP (**Figure S3**). Additionally, although modest heterogeneity was observed between the three *in silico* sorted T_FH_ populations, each tended to occupy different regions at different densities (**Figure S4**). We thus decided to use unsupervised clustering to gain unbiased insight into each of the populations and their relationships with one another. Twelve clusters emerged, several representing canonical CD4^+^ T cell and T_FH_ phenotypes (**Figure 1C**). Select signature markers (**Figure S5**), as well as the heatmap in **Figure 1C** were used to annotate clusters 0-8. Clusters 9-11 were not considered in downstream analyses due to their insignificant sizes. To evaluate the impact of ART on phenotype distribution, we next calculated the frequencies of ART^+^ and ART^-^ patients in each cluster (**Figure S6**). Although some clusters show some trending, we found no significant phenotypic skewing and thus proceeded to group both ART^+^ and ART^-^ patients as HIV^+^ in subsequent analyses of this dataset.

To determine unique features of HIV infection, we compared the distribution of T cells in HIV^+^ and healthy LN-derived cells in each cluster (**Figure 1D**). In agreement the elevated expansion of the T_FH_ compartment seen in the literature (6, 7), we found that cluster 5, denoted as proliferating T_FH_ based on high expression of Ki67 and BLyS, was significantly enriched in HIV^+^ donor LN cells (p < 0.001). Surprisingly, even though cluster 2 had the highest and most homogeneous expression of CXCR3 (**Figure S5**), we found no difference between HIV^+^ and healthy cells. This might reflect that many of the CXCR3^+^T_FH_ in HIV infection take on diverse phenotypic programs. Specifically, given the proximity of cluster 2 with the NAÏVE cluster, it is possible that the CXCR3^+^T_FH_ within cluster 2 represent early T_FH_ entering the LN as previously described (20), whereas CXCR3^+^T_FH_ taking on alternative phenotypic programs may represent other stages of the T_FH_ lifecycle. In line with this argument, we reasoned that cluster 5, in addition to being enriched in HIV^+^ donors, might also have signatures unique to HIV infection. To test this hypothesis, we subsampled cluster 5 to have equal numbers of HIV^+^ and healthy donor cells and ran a likelihood-ratio test on each marker (49). We found that CXCR3 expression in cluster 5 was in fact significantly higher in HIV^+^ than healthy donors (**Figure 1E**). Given the HIV-intrinsic upregulation of CXCR3 in cluster 5, we reasoned that the *in silico* sorted populations might have differential distributions within cluster 5. As expected, CXCR3^+^T_FH_ were significantly enriched in cluster 5 over all other populations in HIV^+^ patients, but not healthy donors (**Figure S7**). Concordantly, CXCR3^+^T_FH_ from HIV^+^ donors occupied cluster 5 at a significantly higher frequency than from healthy donors, whose predominant population was contrastingly CXCR3^-^T_FH_ (**Figure S8**). In addition to CXCR3, Granzyme A, a marker indicative of cytotoxic function in T-helper cells (50), was also enriched in cells from HIV^+^ donors within cluster 5 (**Figure 1E**). It is possible that, although CXCR3^+^T_FH_ generally reside in a more quiescent, immature state in the steady state, HIV infection triggers them to activate, proliferate in the LN, and take on phenotypic signatures unique to HIV infection.

To better understand the relationship between the phenotypic signatures that the *in silico* sorted T_FH_ populations (**Figure S1**) take on specifically in HIV infection, we did pairwise comparisons of the distributions of CXCR3^+^, CXCR3^-^, and CXCR5^-^ T_FH_ populations within HIV+ donors across each cluster (**Figure 1F**). Reflecting the cellular plasticity of T_FH_, gating down on only a handful of markers unsurprisingly revealed modest heterogeneity within each population. As expected, however, cluster 2, defined by the highest expression of CXCR3, bore a significantly large proportion of CXCR3^+^T_FH_ compared to all other populations. In line with previous studies (8, 9), CXCR5^-^T_FH_ were significantly skewed toward activated states in clusters 5 and 6 (Proliferating T_FH_ and EOMES^+^T_FH_, respectively), while CXCR3^+^ and CXCR3^-^T_FH_ both tended to exist in cluster 7 (Activated GC-T_FH_). Although statistical significance was only reached between CXCR3^+^T_FH_ and CXCR3^-^T_FH_ in cluster 2, we noticed that CXCR3^+^T_FH_ often existed at a frequency between CXCR3^-^T_FH_ and CXCR5^-^T_FH_, suggesting this population might exist as a transitional state between the canonical T_FH_ and the recently characterized CXCR5^-^T_FH_. Given this possibility, we next evaluated the median position of each HIV+ donor’s populations on the same UMAP coordinates as in **Figure 1C** (**Figure 1G**). At first glance, the Naïve population is clearly distinct from the T_FH_ populations, which group together on the right side of the UMAP. We also noted that, as suggested in **Figure 1F**, CXCR3^+^T_FH_ appear at an intermediate position between CXCR3^-^T_FH_ and CXCR5^-^T_FH_. Statistical analysis of each population also revealed that all populations, except CXCR3^+^T_FH_ and CXCR5^-^T_FH_, were distinct over UMAP_1 (**Table S3**). Additionally, hierarchical clustering of each population from each donor revealed a grouping of CXCR3^+^T_FH_ with CXCR5^-^T_FH_ more than with CXCR3^-^T_FH_ (**Figure S9**). In concordance with our hypothesis, these data also posit CXCR3^+^T_FH_ as an interim population between CXCR3^-^T_FH_ and CXCR5^-^T_FH_.

### Transcriptomic Analysis Reveals CXCR3^+^ and CXCR3^-^T_FH_ Are Transcriptionally Distinct but Similar on Canonical T_FH_ Marker Genes

Our findings in the Mass CyTOF dataset prompted us to gain deeper insight into the similarities and differences of these T_FH_ populations in HIV infection. Although Mass CyTOF can be useful for delineating cell states due to its ability to analyze large numbers of single cells rapidly and robustly for a selected panel of surface markers, it failed to provide an unbiased analysis of the transcriptome differences between samples. Additionally, due to the destructive nature of Mass CyTOF, TCR sequences, and thus clonal relationships, cannot be obtained. To circumvent these limitations, we sorted five populations of T cells for bulk RNA sequencing (RNA-Seq) from 7 HIV^+^, non-ART-treated donors using the gating strategy illustrated in **Figure S10**. We excluded the one ART-treated donor in this section as we were unsure how treatment would affect a highly sensitive assay such as RNA-seq. We focused specifically on five populations. The first two, CXCR3^+^T_FH_ (CD45RO^+^CXCR5^+^CCR7^-^PD1^+^CXCR3^+^), and CXCR3^-^T_FH_ (CD45RO^+^CXCR5^+^CCR7^-^PD1^+^CXCR3^-^), were sorted to delineate the specific effects of CXCR3 expression on the state of LN T_FH_. GC-T_FH_ (CD45RO^+^CXCR5^+^PD1^+^CD57^+^) and CXCR5^-^T_FH_ (CD45RO^+^CXCR5^-^ICOS^+^PD1^+^) were sorted as two distinct reference points between two subsets that have been demonstrated to provide B cell help (8, 9). CD57-expressing T_FH_ were sorted as a proxy for GC-T_FH_ as they have been shown to be a major subset of active GC cells (51–53), which serves as a positive control for T_FH_ function. An initial analysis on CyTOF and Flow Cytometry data shows comparable expression of CD57 between CXCR3^+^T_FH_ and CXCR3^-^T_FH_ populations (**Figures S11, S12**). Naïve CD4+ T cells CD45RO^-^CXCR5^-^CCR7^+^) were sorted as a non-T_FH_ control.

**Figure 2:**
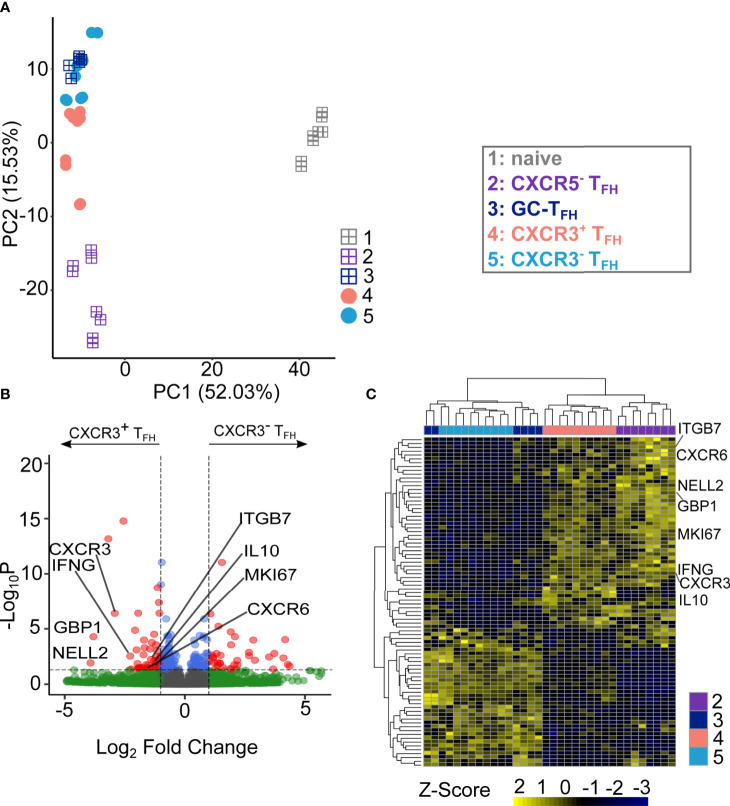
CXCR3^+^T_FH_ population and CXCR3^-^T_FH_ are functionally distinct. **(A)** PCA visualization of the transcriptional data of different T cell subsets. Square-plus dots represent the well-established T cell populations while circle dots represent the T_FH_ subsets. Numbers on the axis represents the proportion of variance. **(B)** Volcano plot of DEGs between CXCR3^+^T_FH_ and CXCR3^-^T_FH_. Highlighted genes are upregulated genes of interest within CXCR3^+^T_FH_ population. DEGs were identified as log2(Fold Change) larger than 1 and adjusted P value (Benjamini-Hochberg adjustment) < 0.05. **(C)** Heatmap showing the expressing of DEGs.

Given a clear enrichment of CXCR3^+^T_FH_ within HIV^+^ patient LNs in the Mass CyTOF dataset, we wanted to understand their transcriptional characteristics in HIV infection. Of particular concern, existing studies with animal LN (27) and human blood (31) show contradicting results on their potential for B cell help. We thus set out to explore the transcriptional landscape of CXCR3^+^T_FH_ in relation to the other, better characterized, T_FH_ populations described above using RNA sequencing (RNA-Seq). We projected the RNA-Seq data from each population onto 2-dimensional space using Principal Component Analysis (PCA). As expected, the naïve population was distinct from all the other populations based on PC1. The T_FH_ populations, although similar on PC1, were separated based on PC2 (**Figure 2A**). In concordance with our analysis on the Mass CyTOF data, CXCR5^-^T_FH_ were distinguishable from GC-T_FH_ and CXCR3^-^T_FH_ on PC2, while CXCR3^+^T_FH_ situated in the middle. These data again suggest CXCR3 expression on T_FH_ may be indicative of a transitional state between the canonical GC-T_FH_ and the recently described motile CXCR5^-^T_FH_ (9). Further advocating for CXCR3^+^T_FH_ as a transitional state, we also noticed CXCR3 expression in both flow cytometry and Mass CyTOF was higher in CXCR5^-^T_FH_ than CXCR5^+^T_FH_ (**Figure S13**). To better understand the transcriptome differences among various T_FH_ populations, we next used Metascape (43) to evaluate the pathways enriched in genes contributing to PC2 (**Figure S14**). Interestingly, genes that mostly negatively correlated with PC2 (upregulated in CXCR5^-^T_FH_ while downregulated in GC-T_FH_) were enriched in cell division and migration. However, genes that most positively correlated with PC2 (upregulated in GC-T_FH_ while downregulated in CXCR5^-^T_FH_) were enriched on pathways inhibiting cell proliferation and migration (**Table S7**). For example, *CTLA4* transmits inhibitory signal to T cells proliferation (54), *SMAD3* mediates inhibition of CD4 T-cell proliferation (55), *MCC* blocks cell cycle progression from G0/G1 to S phase (56). In support of our CyTOF dataset, CXCR3^+^T_FH_ and CXCR5^-^T_FH_ appear to be in a more activated, motile state than their canonical GC-T_FH_ counterpart.

To evaluate the differences between CXCR3^+^ and CXCR3^-^T_FH_ more directly, we next analyzed differentially expressed genes (DEGs, **Figure 2B**) between the two populations. In total, 86 out of 12,515 genes were identified as significant DEGs (**Table S8**). Hierarchical clustering based on those DEGs further demonstrated the similarity of CXCR3^-^T_FH_ with GC-T_FH_ and CXCR3^+^T_FH_ with CXCR5^-^T_FH_ (**Figure 2C**). A significant upregulation of canonical T_H1_ genes (*CXCR3*, *IFNG*, *CXCR6* and *GBP1*) in CXCR3^+^T_FH_ also indicated their unique T_H1_-like program. Additionally, upregulation of *NELL2* and *MKI67* in CXCR3^+^T_FH_ suggested an increased proliferative capacity compared to their CXCR3^-^ counterpart. Interestingly, CXCR3^+^T_FH_ also upregulated *ITGB7*, encoding a subunit of the integrin α4β7, that plays a role in leukocyte adhesion and can serve as a homing receptor bound by HIV (27). Thus, it is possible that CXCR3^+^T_FH_ could be similarly, or even more, susceptible to HIV infection than other T_FH_ subsets. Surprisingly, *IL10*, encoding an unconventional cytokine (IL10) in T_H1_ cells, was also highly expressed by CXCR3^+^T_FH_. Since previous studies support IL10 as a key player in the establishment and perpetuation of HIV persistence (57), the upregulation of *IL10* in CXCR3^+^T_FH_ may result in enhanced persistence of HIV after infection.

To infer the functional potential of human LN-derived CXCR3^+^T_FH_, we next compared them to CXCR3^-^T_FH_ specifically on T_FH_-related genes. We created a T_FH_ signature gene set using previously published RNA-Seq data from human tonsil samples (31). Both CXCR3^+^T_FH_, CXCR3^-^T_FH_ and GC-T_FH_ appeared similar when hierarchically clustered on tonsil T_FH_ signature genes (**Figure S15A**). GSEA and GSVA based on the same gene set also revealed no significant differences between these two cell populations (**Figures S15B, C**). Detailed analysis on several manually curated T_FH_-related genes also suggested CXCR3^+^ and CXCR3^-^T_FH_ bear similar T_FH_ marker gene expression patterns (**Figure S15D**). For example: *FOXO1*, a negative regulator of *BCL6 (* 58), was downregulated in both populations. Additionally, *BCL6*, *MAF* and *CD84*, key transcriptional regulators of T_FH_ differentiation, were similar in both CXCR3^+^ and CXCR3^-^T_FH_. *B3GAT1* (an enzyme necessary for the production of *CD57*), which is specifically expressed by active GC-T_FH_ (8, 51–53), was also comparably expressed in both populations. As expected, CXCR5 and its upstream regulator *ASCL* were both similarly expressed in CXCR3^+^ and CXCR3^-^T_FH_. Taken together, these observations reveal that CXCR3^+^T_FH_ have a similar T_FH_ transcriptional program to CXCR3^-^T_FH_, suggesting a functional overlap between the two populations.

In summary, global transcriptome analysis depicts CXCR3^+^T_FH_ unique from GC-T_FH_, biasing toward a T_H1_-like program. It also revealed CXCR3^+^T_FH_ are similar to the recently described CXCR5^-^T_FH_. Focusing specifically on T_FH_ marker genes, significant differences between CXCR3^+^ and CXCR3^-^ T_FH_ were not observed, supporting the paradigm that human LN-derived CXCR3-expressing T_FH_ may still bear T_FH_ function.

### CXCR3^+^T_FH_ Upregulate Cell Migratory Pathways and Uniquely Express a Signature of CXCR5^-^T_FH_

**Figure 3:**
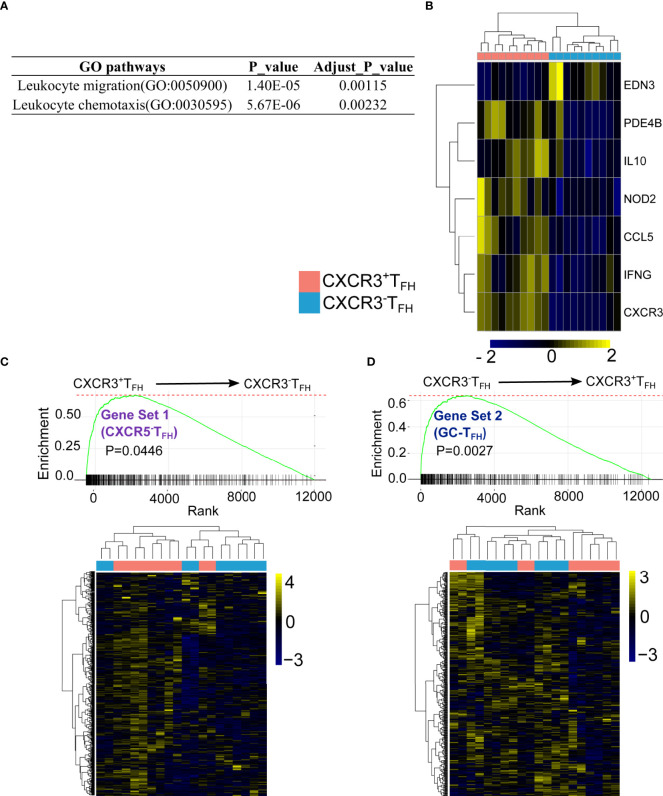
CXCR3^+^T_FH_ uniquely express cell migratory genes and transcriptomic-ally similar with CXCR5^-^T_FH_. DEGs between CXCR3^+^T_FH_ and CXCR3^-^T_FH_ were enriched on T cell migration pathways **(A)** with migratory genes upregulated in CXCR3^+^T_FH_ (Leukocyte chemotaxis, GO:0030595) **(B)** GSEA analysis indicates genes elevated in CXCR5^-^T_FH_ (Gene set 1) were upregulated in CXCR3^+^T_FH_ **(C)** while genes upregulated in GC-T_FH_ (Gene set 2) were also elevated in CXCR3^-^T_FH_ **(D)** GSEA were performed with R package ‘fgsea’, specifically, genes were pre-ranked by their p-values, which indicates whether one gene is highly expressed in CXCR3^+^T_FH_ or CXCR3^-^T_FH_. The bar on the X-axis indicates one gene from a selected gene set (i.e., either Gene set 1 or Gene set 2). The curve is a running sum of the bars on the X-axis.

To investigate deeper into the functional program of CXCR3^+^T_FH_, we used NetworkAnalyst (44) to identify GO biological pathways enriched within this population. Only two GO pathways were significantly enriched within CXCR3^+^T_FH_ when compared with CXCR3^-^T_FH_. Interestingly, both pathways were related to T cell migration (**Figure 3A**). We visualized the differentially expression genes of these two pathways in detail, as expected, most of the genes were upregulated in CXCR3^+^T_FH_ (**Figure 3B**; **Figure S16**), which suggested a possibility of this cell subset to be more motile.

Our previous study (9) suggested that T_FH_ can downregulate CXCR5 expression and accumulate as CXCR5^-^T_FH_ in LNs during HIV infection. The same study also found that these CXCR5^-^T_FH_ provide B cell help and have a propensity to migrate into the periphery. The combination of CXCR3^+^T_FH_ also bearing a cell migration signature, as well as existing in an intermediate cell state between CXCR5^-^T_FH_ and GC-T_FH_ based on both Mass CyTOF and RNA-Seq data, led us to hypothesize that CXCR3^+^T_FH_ may be the immediate relative of CXCR5^-^T_FH_. To test this hypothesis more directly, we compared the transcriptomes of CXCR3^+^ and CXCR3^-^T_FH_ using GC-T_FH_ and CXCR5^-^T_FH_ as reference gene sets. Specifically, we first identified 938 DEGs from 12,559 total shared genes between GC-T_FH_ and CXCR5^-^T_FH_. Among these DEGs, 514 were upregulated in CXCR5^-^T_FH_ (Gene set 1) and 424 were upregulated in GC-T_FH_ (Gene set 2). We then performed GSEA analysis to compare CXCR3^+^ and CXCR3^-^T_FH_ on Gene Set 1 and 2 (**Figures 3C, D**). As hypothesized, CXCR3^+^T_FH_ followed the CXCR5^-^T_FH_ program, while CXCR3^-^T_FH_ more closely followed the GC-T_FH_ program.

Together with our observations in Mass CyTOF, global transcriptome, differential gene expression, gene set enrichment, and pathway analyses, we conclude that CXCR3^+^T_FH_ likely exist in a phenotypically intermediate state between canonical GC-T_FH_ and the more motile and peripheral CXCR5^-^T_FH_.

### T Cell Receptor Repertoire Demonstrates That CXCR3^+^T_FH_ Are Clonally Related to CXCR5^-^T_FH_ and Peripheral T_FH_

After observing that CXCR3^+^T_FH_ upregulated cell migratory pathways and skew phenotypically toward CXCR5^-^T_FH_, we suspected that changes in CXCR3 expression on T_FH_ might facilitate transitions to and from GC-T_FH_ and CXCR5^-^T_FH_ cell states. To test this hypothesis, we used bulk T cell receptor (TCR) repertoire sequencing to measure their *in vivo* clonal relationship with various T cell populations. Because of the immense diversity generated by V(D)J recombination, TCR sequences can be thought as unique ‘ID-Cards’ (59), where cells sharing a given TCR sequence are likely to be the progenies of the same ancestor. By comparing the overlap of TCR sequences among samples, a clonal lineage across tissues and cell states can be inferred.

**Figure 4:**
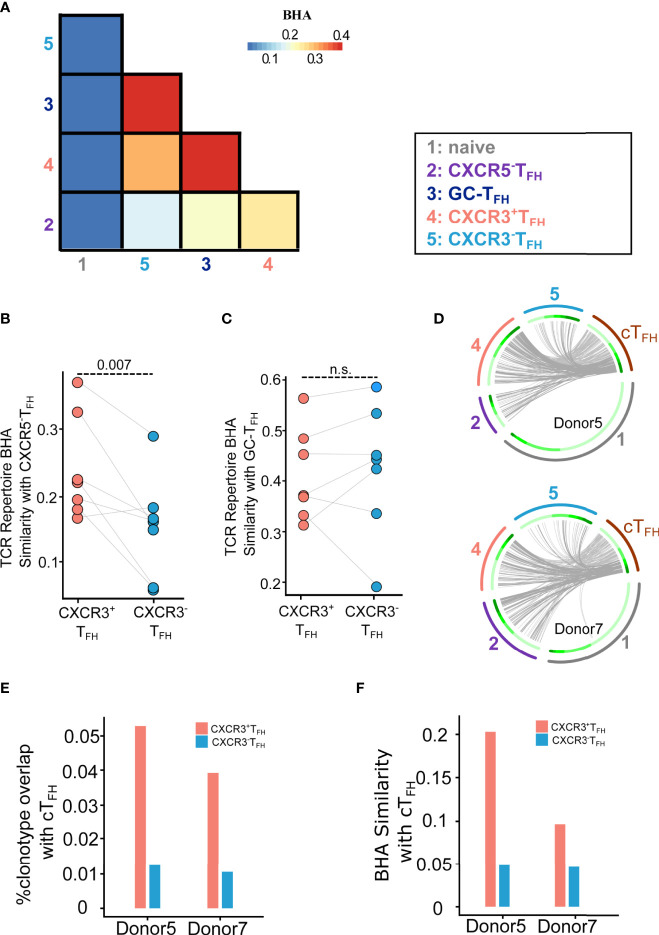
TCR repertoire analysis demonstrates the clonal relationship of CXCR3^+^T_FH_ with the CXCR5^-^T_FH_ outside GC and cT_FH_ in the blood. **(A)** Average BHA similarity among different T cell subsets. **(B)** Dot plot shows BHA similarity of CXCR3^+^/CXCR3^-^T_FH_ with the CXCR5^-^T_FH_ population (n = 7). **(C)** Dot plot shows BHA similarity comparing CXCR3^+^/CXCR3^-^T_FH_ with GC-T_FH_ (n = 7); n.s.: not significant. **(D)** Circos plots show the overlap of cT_FH_ TCR with LN T cell populations. Two donors were analyzed. Each small slice of the arc represents one TCR clonotype, sorted by its size (darker green for larger clones, inner circle). The gray curves link overlapping TCR nucleotide clonotypes in naïve (black, outer circle), CXCR5^-^T_FH_ (dark blue, outer circle), CXCR3^+^T_FH_ (blue, outer circle), CXCR3^-^T_FH_ (red, outer circle) and cT_FH_ (dark green, outer circle). The most expanded 30% clonotypes were highlighted with darker gray curves. **(E)** Histogram plot shows percentage of TCR clonotype overlapping comparing CXCR3^+^/CXCR3^-^T_FH_ with cT_FH_. **(F)** Histogram plot shows BHA similarity comparing CXCR3^+^/CXCR3^-^T_FH_ with cT_FH_.

We thus compared the TCR repertoire similarity across the five LN T cell populations using Bhattacharyya Coefficient (36) as the similarity index. Bhattacharyya coefficient (BHA) measures the overlap of clonotypes between two T cell populations, while taking size of clones into consideration. In contrast to previous findings in healthy human tonsils (23), CXCR3^+^T_FH_, CXCR3^-^T_FH_ and GC-T_FH_ were strikingly similar in their repertoires across all donors (**Figures 4A, C**; **Figure S17**). This suggests that these three populations are likely capable of transitioning in and out of each of these states with ease, which may be a phenomenon intrinsic to HIV infection. In concordance with transcriptomic data, however, CXCR3^+^T_FH_ were significantly more related to CXCR5^-^T_FH_ than CXCR3^-^T_FH_ across all donors (**Figures 4A, B**). For two out of the seven donors, we were able to compare the CXCR3^+^ and CXCR3^-^T_FH_ repertoires with the cT_FH_ repertoires in the blood (60) (**Figures 4D–F**; **Figure S18**). Circos plots representing the overlap of TCRs between cT_FH_ and LN populations indicate that CXCR3^+^T_FH_ are also more clonally related to cT_FH_ than their CXCR3^-^T_FH_ counterpart (**Figure 4E**), similar to a recent finding in healthy tonsils (23). A similar trend was observed considering clone size weighted BHA index (**Figure 4F**). In combination, high clonal overlap amongst all CXCR5-expressing T_FH_ in the LN, and the elevated relationship of CXCR3^+^T_FH_ with CXCR5^-^T_FH_ and cT_FH_, suggests that this population sits as a bridge between secondary lymphoid tissue and the periphery.

### CXCR3^+^T_FH_ in the Human LN Are a Reservoir for HIV

T_FH_ are major reservoirs for HIV (61), complicating their role in controlling virus through the coordination of GC reactions. Furthermore, CXCR3^+^T_FH_ have been speculated to maintain a dynamic HIV reservoir due to high expression of HIV co-receptors (i.e. CCR5 and α4β7) (62). High viral load in this highly motile cell type could either exacerbate systemic spread of virus or expose viral antigens to the immune system. Given our result that CXCR3^+^T_FH_ frequency negatively correlates with pVL (**Figure 1C**), we speculated a high level of HIV copies in CXCR3^+^T_FH_ might be a mechanism for improving clearance of virus.

**Figure 5:**
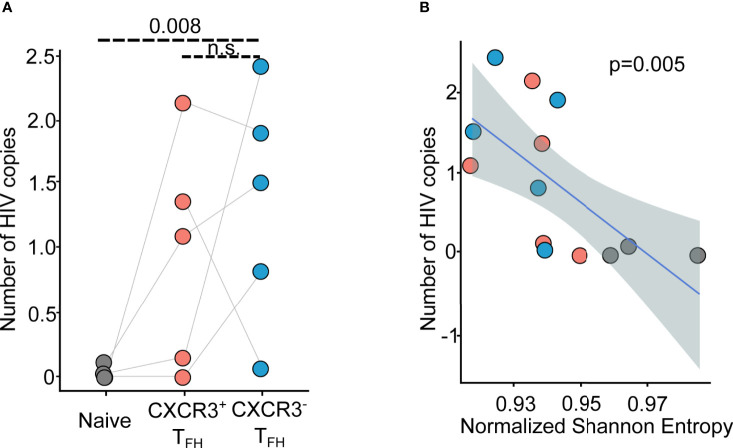
HIV transcript mapping shows CXCR3^+^T_FH_ is one major reservoir of HIV viruses. **(A)** Dot plot shows the distribution of normalized HIV copy number across donors and T cell populations (n = 5); n.s.: not significant; **(B)** Scatter plot shows the correlation between normalized HIV copy number versus Normalized Shannon Entropy (NSE). Blue line represents the regression line, while the dashed area shows the 95% confident interval.

To investigate this idea, we mapped the bulk RNA-Seq reads from each T cell population to the HIV genome to quantify their relative HIV infection intensities (see methods for details). We detected comparable levels of HIV transcripts in both CXCR3^+^ and CXCR3^-^T_FH_, although both were significantly higher than Naïve cells (**Figure 5A**; **Figure S21**). Similar to experiments in RMs (27), HIV abundance also negatively correlated with repertoire diversity (**Figure 5B**), reflecting the propensity of proliferating and antigen-experienced cells to bear a higher HIV burden. Given that the two T_FH_ subsets discussed here share a similarly high HIV burden, the consequence of the migratory potential of CXCR3^+^T_FH_ is likely a complex balance between limiting systemic spread of virus and exposing it to novel immune compartments.

## Discussion

T_FH_ are paramount to the elicitation of BNAbs. Whether induced by vaccination or natural infection, BNAbs targeting invading pathogens have been shown in a variety of contexts to mitigate, eliminate, and even prevent disease (63, 64). Some viruses, however, can escape from or disrupt this response, often leading to uncontrolled viral replication and chronic infection. In HIV infection, natural BNAbs are seldom produced and almost never lead to viral control (4, 5). Given that T_FH_ are a major site of HIV infection (11–13), it is possible that alternative phenotypic and functional programs within the T_FH_ compartment are taken on to overcome this deficit in humoral immunity. Unfortunately, sampling T_FH_ from humans in their native lymphoid tissue in untreated HIV infection poses an immense challenge in the field. Here, we have overcome this obstacle, combining several high-throughput assays and analyses on T_FH_ from HIV^+^ patient LNs to gain deeper insight into their role in disease.

Mounting evidence points to a high degree of cellular plasticity within the T_FH_ compartment (15–17). We show here the inflation of CXCR3^+^T_FH_ in HIV^+^ patient derived LNs compared to healthy donors that correlates with a lower viral load. Further investigation into this population revealed its abnormal cell state specifically within HIV+ donors that positions it in an intermediate phenotypic state between GC-T_FH_ (8, 31) and the recently described CXCR5^-^T_FH_ (9). Deeper analysis into the clonal relationship of CXCR3^+^T_FH_ corroborated these results, revealing its heightened repertoire similarity with both CXCR5^-^T_FH_ and cT_FH_. Additionally, transcriptome analysis revealed a propensity for CXCR3^+^T_FH_ to upregulate cell migration pathways. Taken together, we posit CXCR3^+^T_FH_ as a bridge between lymphoid tissue and the periphery. Given that these CXCR3^+^T_FH_ bear a high HIV burden, may be primed for cellular migration, and are affiliated with a lower viral load, it is possible that their movement in and out of lymphoid tissues leads to better viral control by exposing antigens to novel immune compartments. If so, it is possible that the upregulation of CXCR3 in T_FH_ may be an effort of the immune system to drive viral reservoirs out of hiding. Given the propensity of low levels of HIV to remain through ART (12) and the increased likelihood that BNAbs are produced during high antigen exposure (10), a mass exodus of T_FH_ may be beneficial to the host during early infection or ART. Furthermore, targeting these migration pathways has the potential to be a viable therapeutic. Of course, it is also possible CXCR3 upregulation in T_FH_ is not HIV-intrinsic, but rather a product of chronic infection. In fact, previous research has implicated CXCR3^+^T_FH_ in HCV infection (60), Zika virus infection (65), and acute febrile malaria (66) and vaccination (67) in children. Interestingly, however, while CXCR3^+^T_FH_ are affiliated with neutralizing antibodies in HCV and Zika virus infection, they appear to lead to poor prognosis in malaria. Understanding the differences and similarities between CXCR3^+^T_FH_ in each of these disease contexts, specifically in lymphoid tissues as addressed in this study, will shed light on different immune-intrinsic properties of chronic infection.

An important limitation of this study was our inability to directly measure T_FH_ function on the populations of interest. Although we were able to point to specific genes, pathways, and signatures that suggest their ability to provide B cell help, definitive knowledge of the functional capacity of CXCR3^+^T_FH_ in human LNs will be important to understanding their role in HIV infection. Future studies should aim at accomplishing this. Additionally, the spectral limitations of FACS also prevented us from being able to sort more specific T_FH_ subsets that may be important to resolve the unique phenotypic programs of CXCR3^+^T_FH_ in HIV infection. For example, our analyses on the Mass CyTOF data suggested that CXCR3^+^T_FH_ take on a unique proliferative program specific to HIV^+^ patients that was not robustly seen in healthy donors, a phenomenon that may explain some of the repertoire discrepancies between this study and others (20). However, since we were unable to accommodate more markers when sorting for TCR and RNA sequencing, especially with the limited number of cells within each LN sample, these unique HIV-intrinsic factors may have been diluted. Recent innovations in antibody barcoding and single cell RNA-seq coupled with creative approaches to isolate these unique T_FH_ populations may lead to a clearer understanding of their phenotypic and clonal relationships.

In summary, we have evaluated several LN-blood-matched T_FH_ from a small, albeit rare cohort of untreated HIV^+^ patients using Mass CyTOF and RNA and bulk TCR repertoire sequencing. Our analyses revealed a phenotypic shift of CXCR3^+^T_FH_ from a GC-T_FH_ cell state toward an unconventional CXCR5^-^T_FH_ state. CXCR3^+^T_FH_ also upregulated migratory transcriptional programs and were clonally related to peripheral T_FH_ cell populations. Altogether, these data suggest that CXCR3^+^T_FH_ may be transitional state between their CXCR3^-^ lymphoid and peripheral counterparts. Future work aimed at delineating the temporal relationships of these T_FH_ populations, drilling deeper into the function of more specific phenotypic niches, will be pertinent to fully understanding their implication in HIV infection.

## Data Availability Statement

Publicly available datasets along with new sequencing data were analyzed and can be found here: https://www.ncbi.nlm.nih.gov/projects/gap/cgi-bin/study.cgi?study_id=phs001548.v2.p1.

## Ethics Statement

Subjects included in this study are from a subset of patients recruited for our previous study (8). All samples were de-identified and obtained with IRB regulatory approval from the University of Pennsylvania. The patients/participants provided their written informed consent to participate in this study.

## Author Contributions

CH and MJM analyzed data, performed research, and wrote the manuscript; MJM, BSW and K-YM performed sequencing experiments; DDA performed cell sorting experiments; DBW and PLDJ helped with data interpretation; PMDR-E, YA-T, and GR-T established the infrastructure to recruit HIV^+^ patients, provided HIV-infected samples, and associated clinical information. NJ and LFS designed the study; NJ directed the study. All authors contributed to the article and approved the submitted version.

## Funding

This work was supported by NIH grants S10OD020072 (NJ), R56AG064801 (NJ), R01AI134879 (LFS), NIH IPCAVD grant U19 Al109646-04(DBW), Chan Zuckerberg Initiative Neurodegeneration Challenge Network Ben Barres Early Career Acceleration Awards 191856 (NJ), VA Merit Award IMMA-020-15F (LFS), NIH/NIAID Collaborative Influenza Vaccine Innovation Centers (CIVICs) contract 75N93019C00051(DBW). DBW is the W.W. Smith Charitable Trust Professor at the Wistar Institute.

## Conflict of Interest

NJ is a Scientific Advisor and holds equity interest in ImmuDX, LLC, and Immune Arch, Inc. In the interest of full disclosure, DBW reports that he serves on Advisories for AstraZeneca, Geneos, Advaccinepharma, he participates in BOD service for Inovio. Remuneration received by DBW for these services includes SRA funding, direct payments, stock or stock options disclosed here.

The remaining authors declare that the research was conducted in the absence of any commercial or financial relationships that could be construed as a potential conflict of interest.

## Publisher’s Note

All claims expressed in this article are solely those of the authors and do not necessarily represent those of their affiliated organizations, or those of the publisher, the editors and the reviewers. Any product that may be evaluated in this article, or claim that may be made by its manufacturer, is not guaranteed or endorsed by the publisher.
