# Supplementary material for: Transcriptome and TCR Repertoire Measurements of CXCR3+ T Follicular Helper Cells Within HIV-Infected Human Lymph Nodes
Source: Front Immunol. 2022 May 6;13:859070. doi: 10.3389/fimmu.2022.859070 (PMC9128546; doi:10.3389/fimmu.2022.859070)
Supplement: Supplementary file 1 [file DataSheet_1.docx]

**SUPPLEMENTARY**


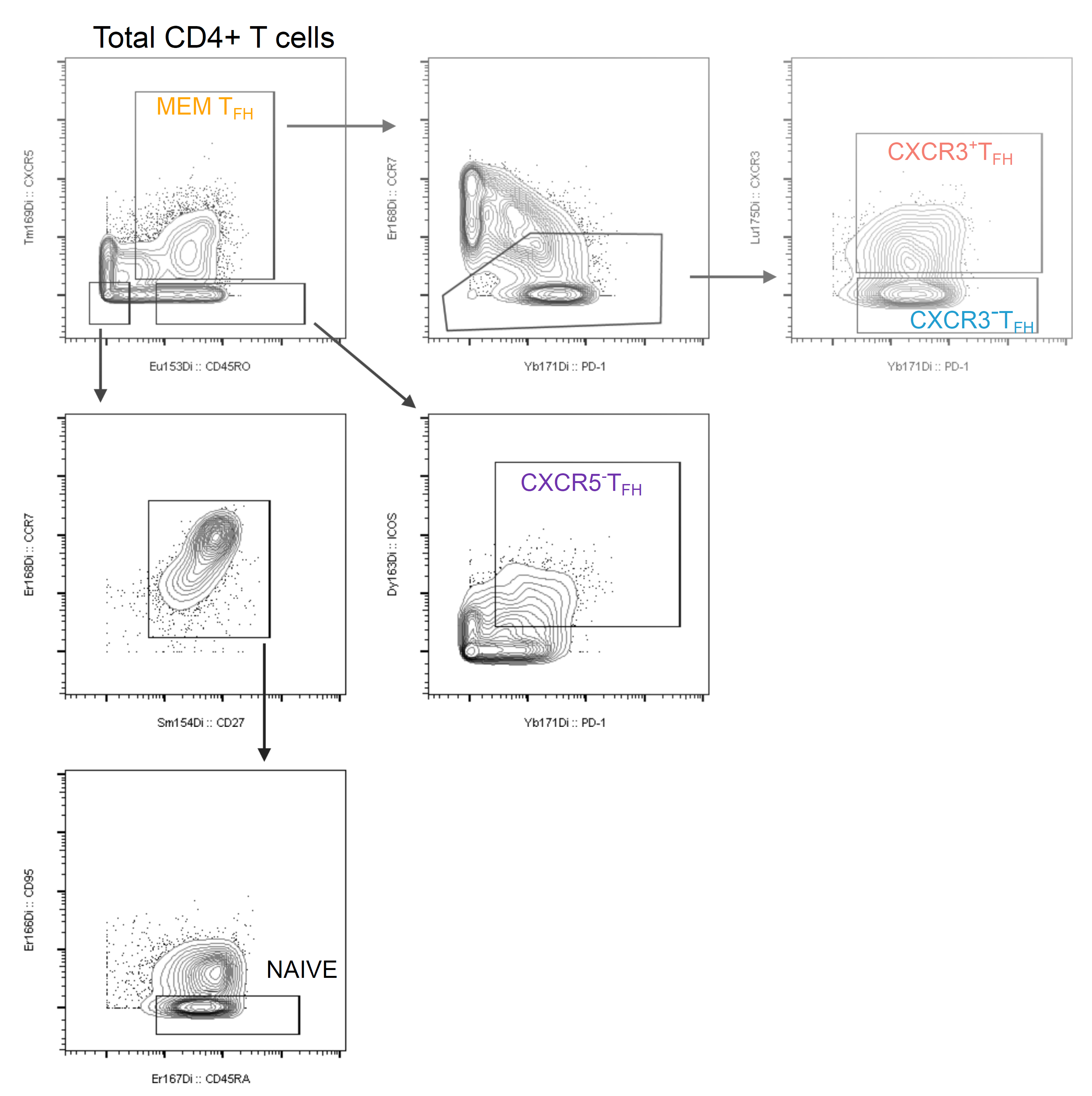


**Figure S1. Gating strategy of MASS CyTOF data**


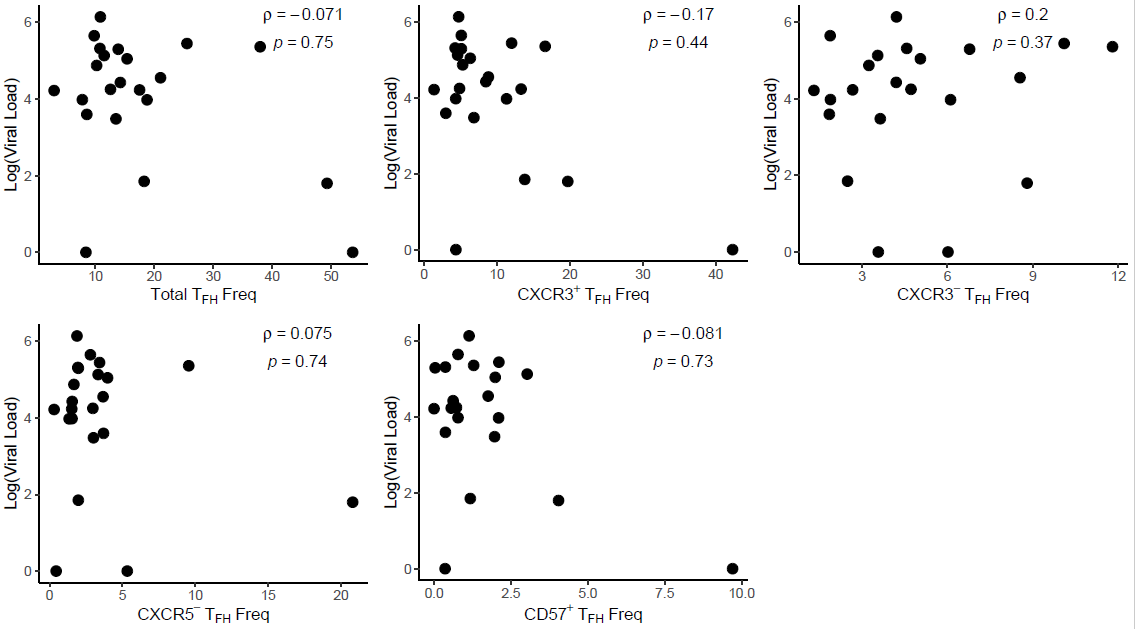


**Figure S2. Correlation of Log pVL versus various TFH population frequencies from Mass CyTOF donors. Spearman’s rank correlation coefficient was used to compare variables.**

**
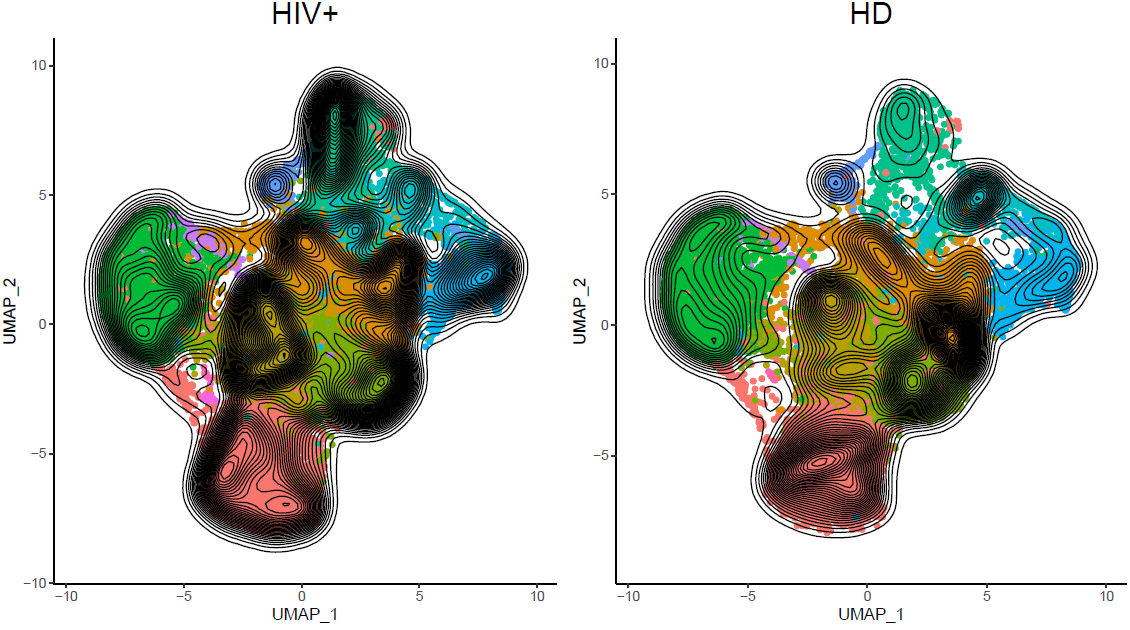
**

**Figure S3. HIV^+^ and HD occupy unique regions within UMAP dimensions.**

**
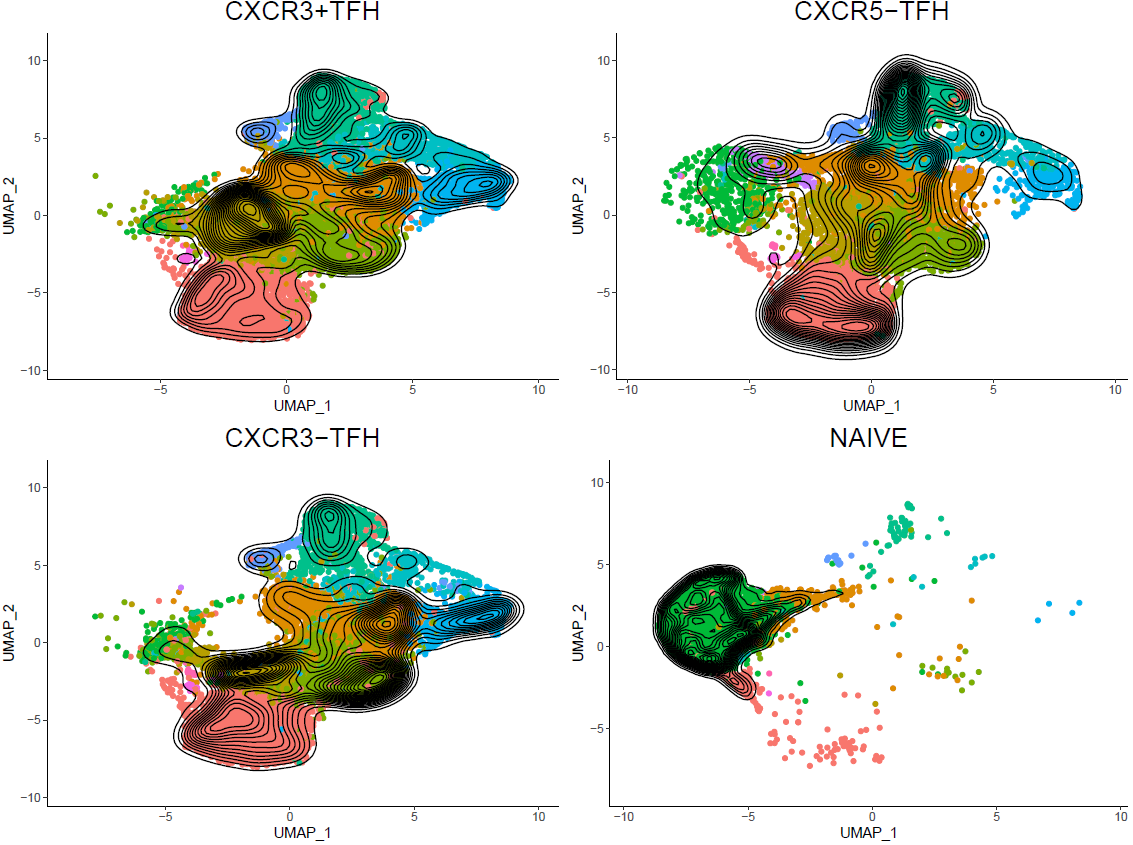
**

**Figure S4. *In Silico* sorted populations occupy unique regions within UMAP dimensions.**

**
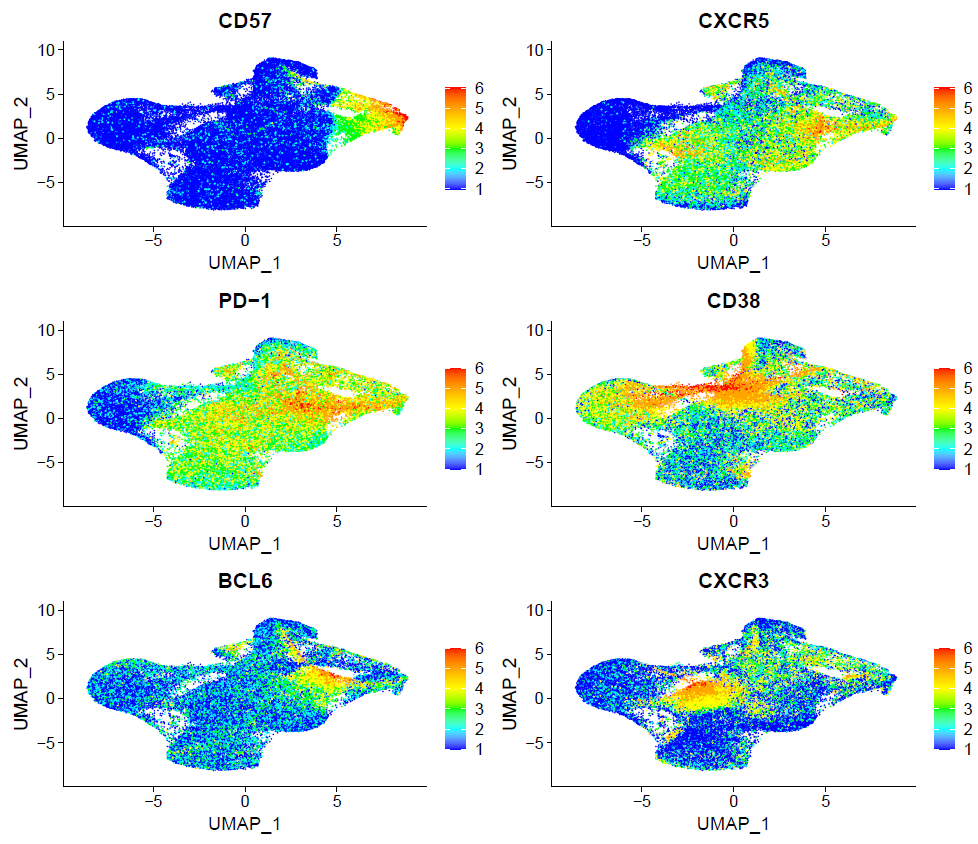
**

**Figure S5. Relative expression of select marker genes used for annotation of clusters in Figure 1B.**


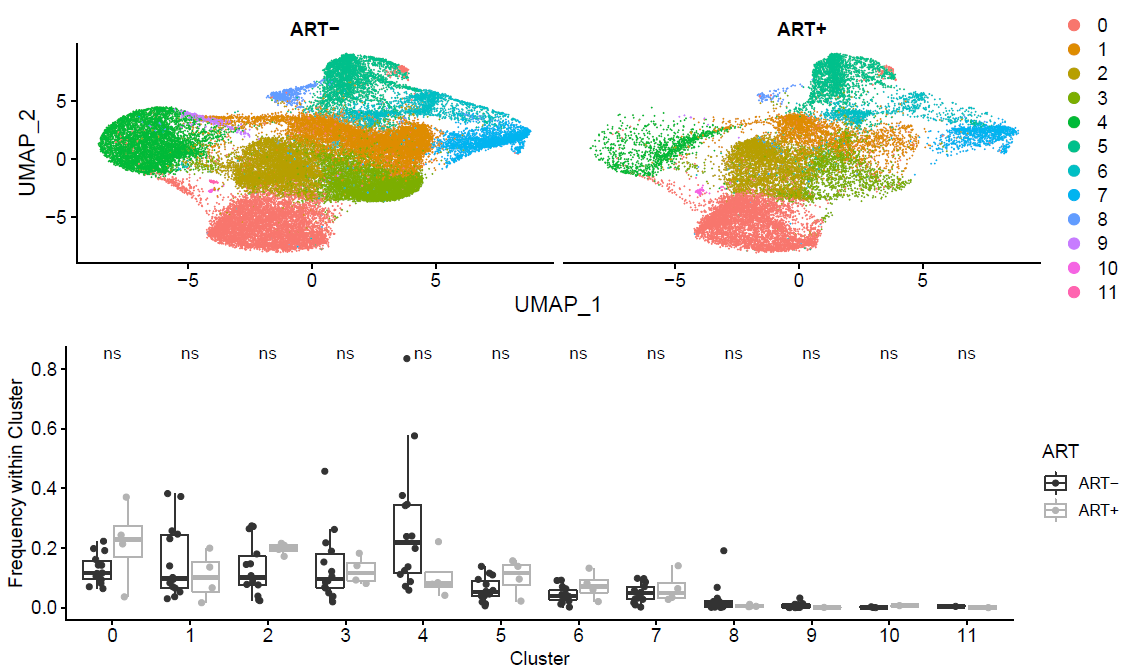


**Figure S6. ART has no apparent effect on cluster distribution in Mass Cytometry.** Frequencies of each population were compared using a pairwise T-test (bottom). Significance: ****: p < 0.0001; **: p < 0.001; **: p < 0.01; *: p <0.05; ns: p ≥ 0.05.

**
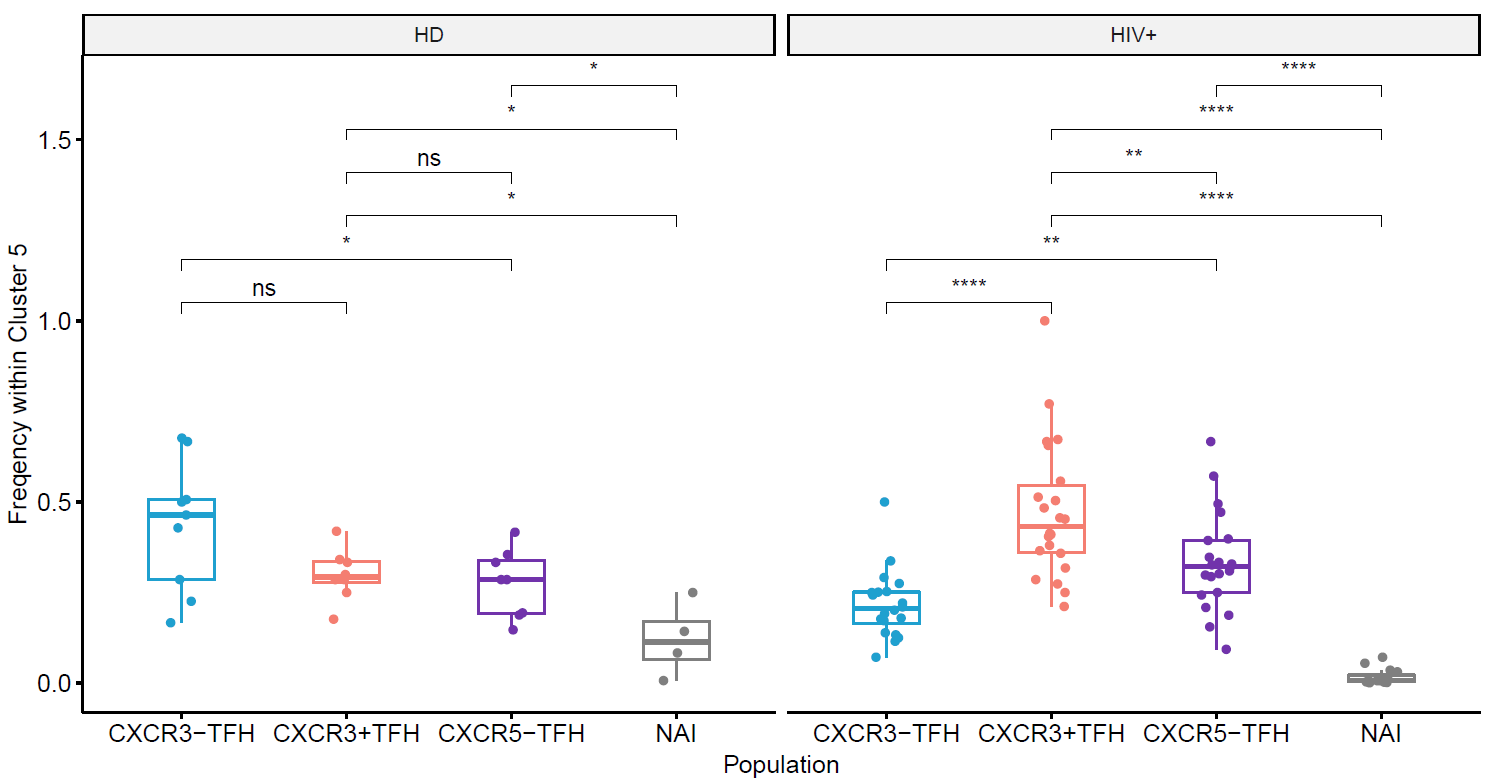
**

**Figure S7. Enrichment of CXCR3^+^T_FH_ within cluster 5 is unique to HIV infection.** Frequencies of each population were compared using a pairwise T-test. Significance: ****: p < 0.0001; **: p < 0.001; **: p < 0.01; *: p <0.05; ns: p ≥ 0.05.

**
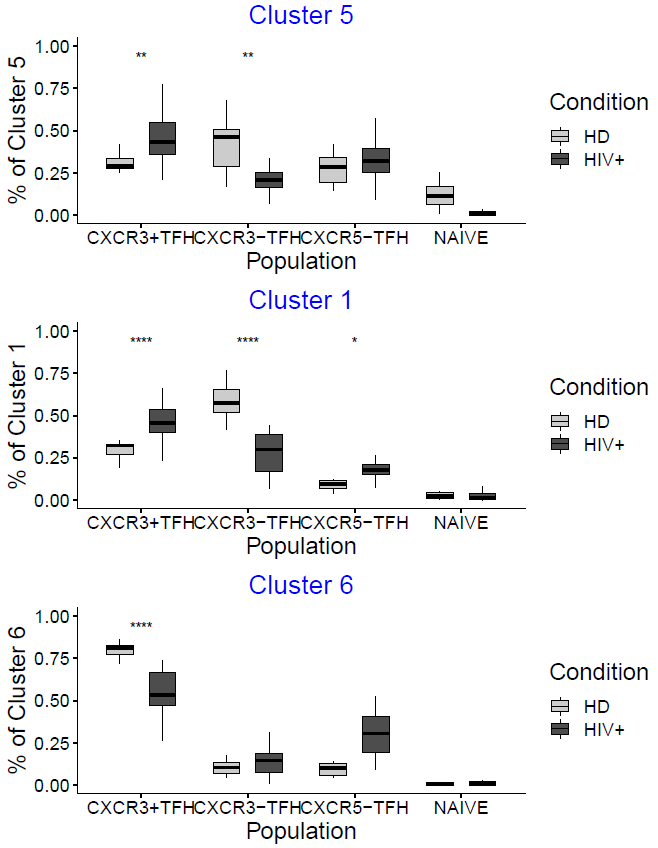
**

**Figure S8. Cluster 5 contains a differential distribution of CXCR3^+^ and CXCR3^-^T_FH_ within HIV infection compared to steady-state.** Frequencies of each population were compared using a pairwise T-test**.** Significance: **: p < 0.01.

**
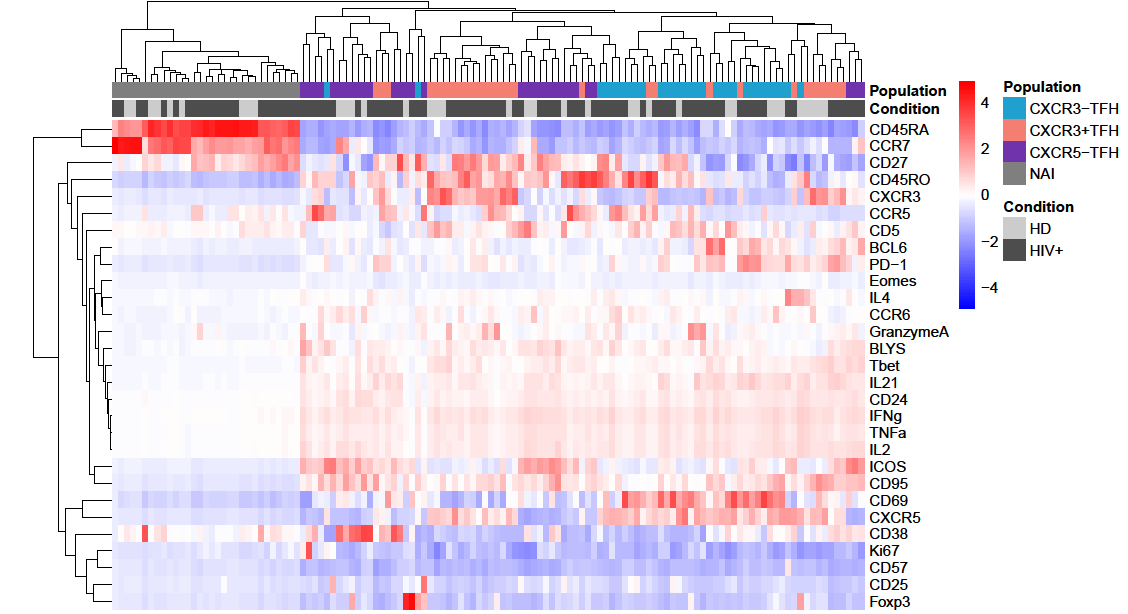
**

**Figure S9. CXCR3^+^T_FH_ and CXCR5^-^T_FH_ hierarchically cluster together on expression Mass CyTOF markers.**

**Figure S10. Characterization of different CD4+ T cell populations sorted.** (A). Representative flow cytometry plots used for identification of T cell subsets. (B). Representative histogram plot of T_FH_ that expressing CXCR3 or not, two distinct clusters of T_FH_ population were observed. (C). Dot plot shows percentage of CXCR3^+^T_FH_ versus CXCR3^-^T_FH_ subsets within T_FH_ (n=7).

**
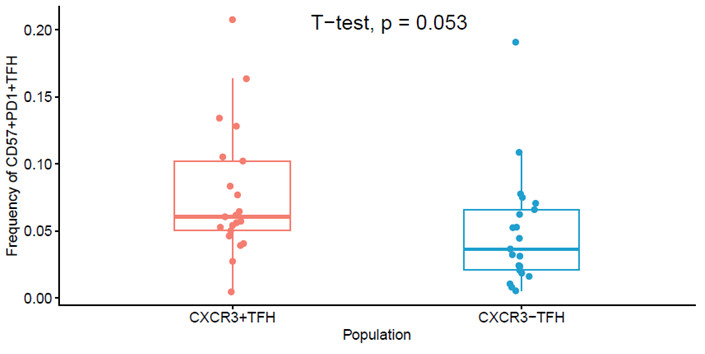
**

**Figure S11. CyTOF analysis shows CXCR3^+^T_FH_ and CXCR3^-^T_FH_ have comparable contribution to *In silico* sorted CD57+PD1+TFH (GC-TFH).**

**
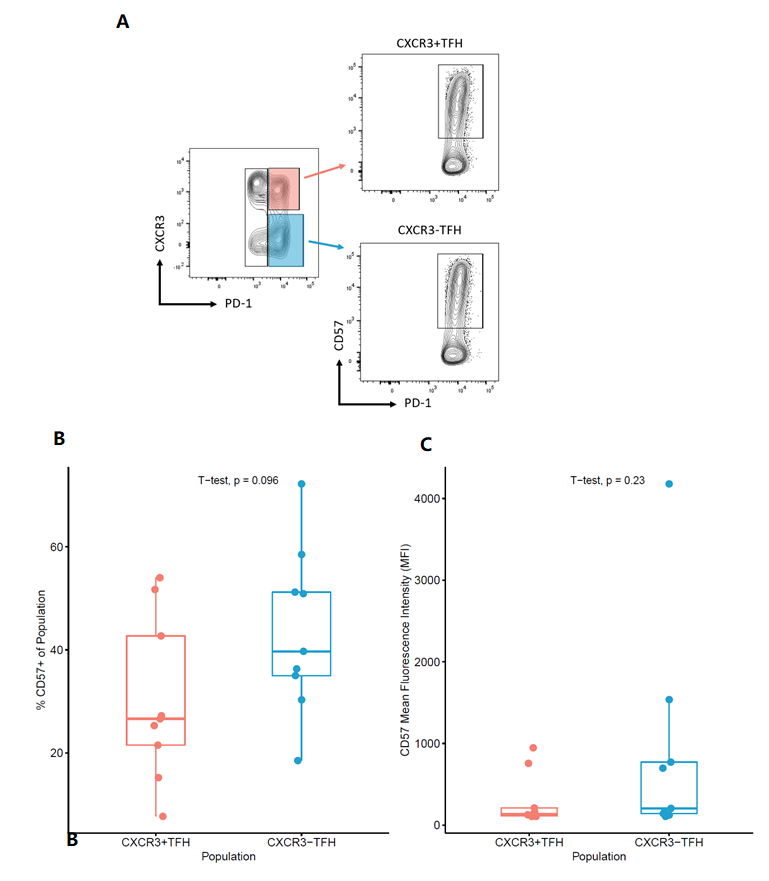
**

**Figure S12. Flow Cytometry analysis shows CXCR3^+^T_FH_ and CXCR3^-^T_FH_ have comparable contribution to CD57+PD1+TFH (GC-TFH).** (A). Representative plots for CXCR3^+^T_FH_ and CXCR3^-^T_FH_ populations sorted as in Figure S9. Populations were drilled down on and compared with respect to frequency (B) and mean fluorescence intensity (C).


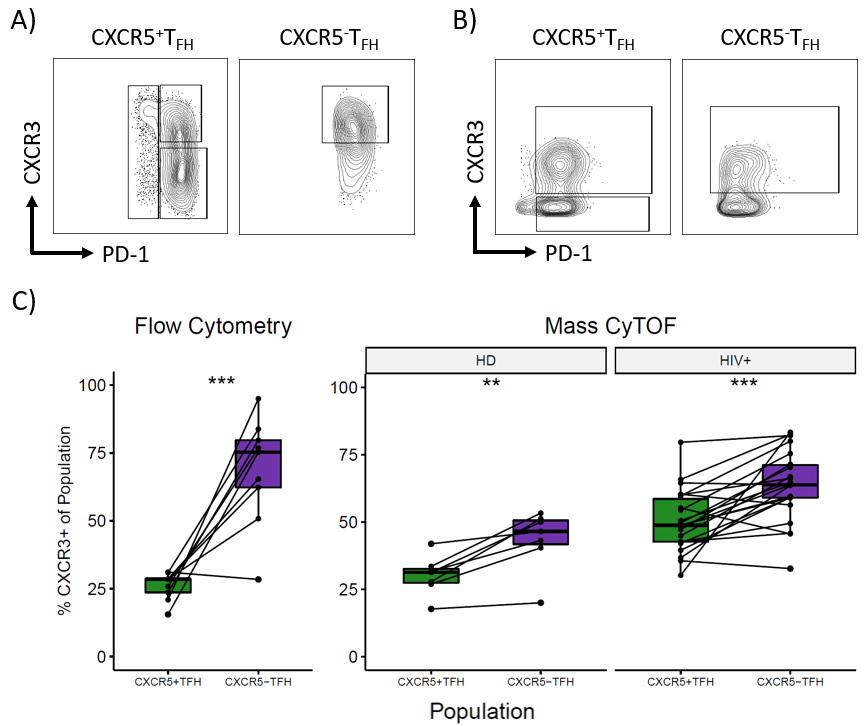


**Figure S13. CXCR5^-^T_FH_ have a larger CXCR3^+^ population than CXCR5^+^T_FH_.** (A). Representative flow cytometry plots from CXCR5^+^ and CXCR5^-^T_FH_ on CXCR3 expression. Prior gates were the same as Fig. S1 (B). Same as A, but from the Mass CyTOF dataset. (C). Summary of the frequencies of CXCR3-expressing T_FH_ in both flow cytometry and Mass CyTOF datasets. P-values were calculated using a paired t-test. Significance: ****: p < 0.0001; **: p < 0.001; **: p < 0.01; *: p <0.05; ns: p ≥ 0.05.


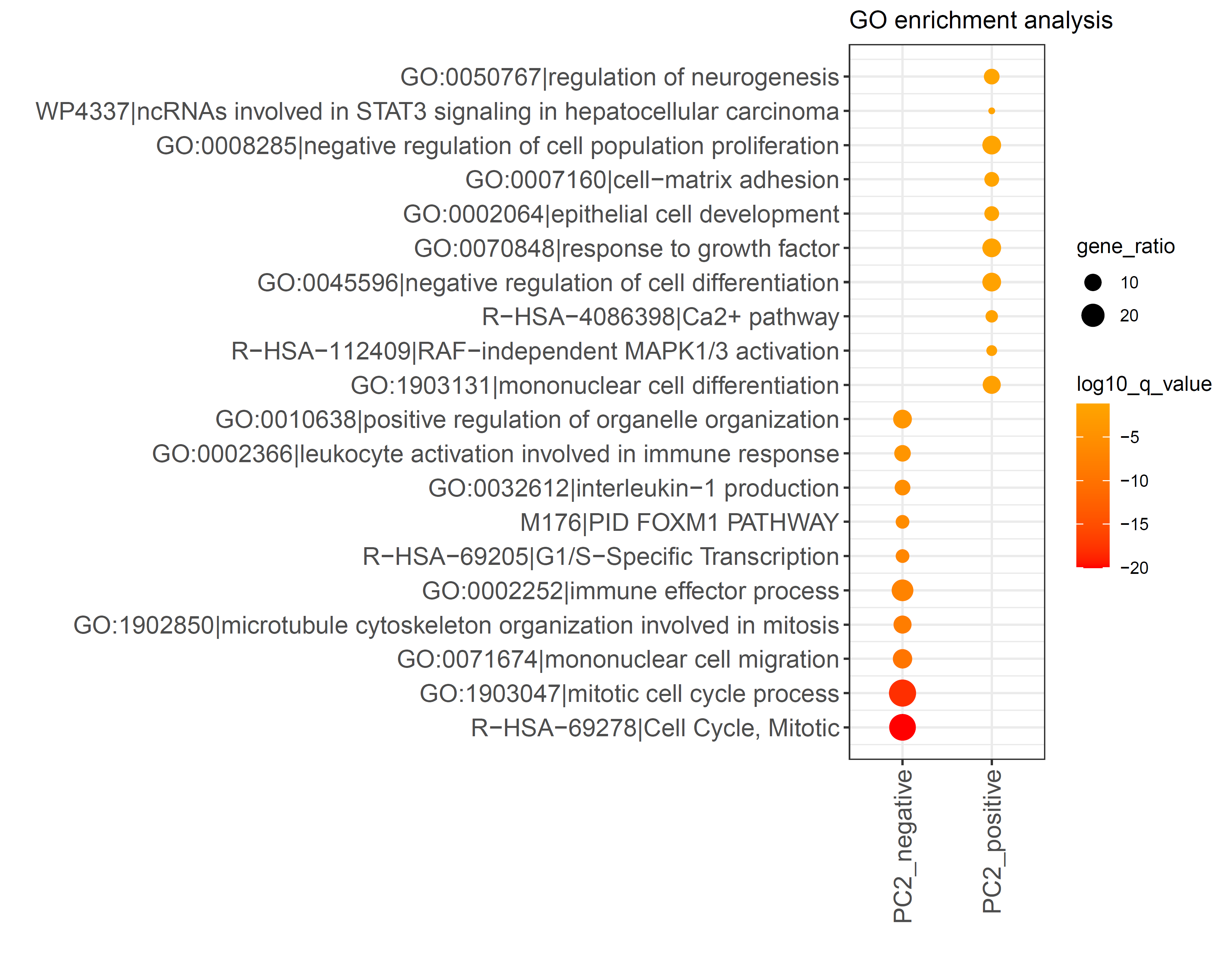


**Figure S14. Pathway enrichment based on the top 100 most positive and negative PC2-correlated genes as determined by Metascape.** Top 10 pathways for each category were plotted. Pathways were ordered by adjust P value (shown by dot color). Dot sizes indicate the percentage of genes in the list that can be mapped to the pathway.


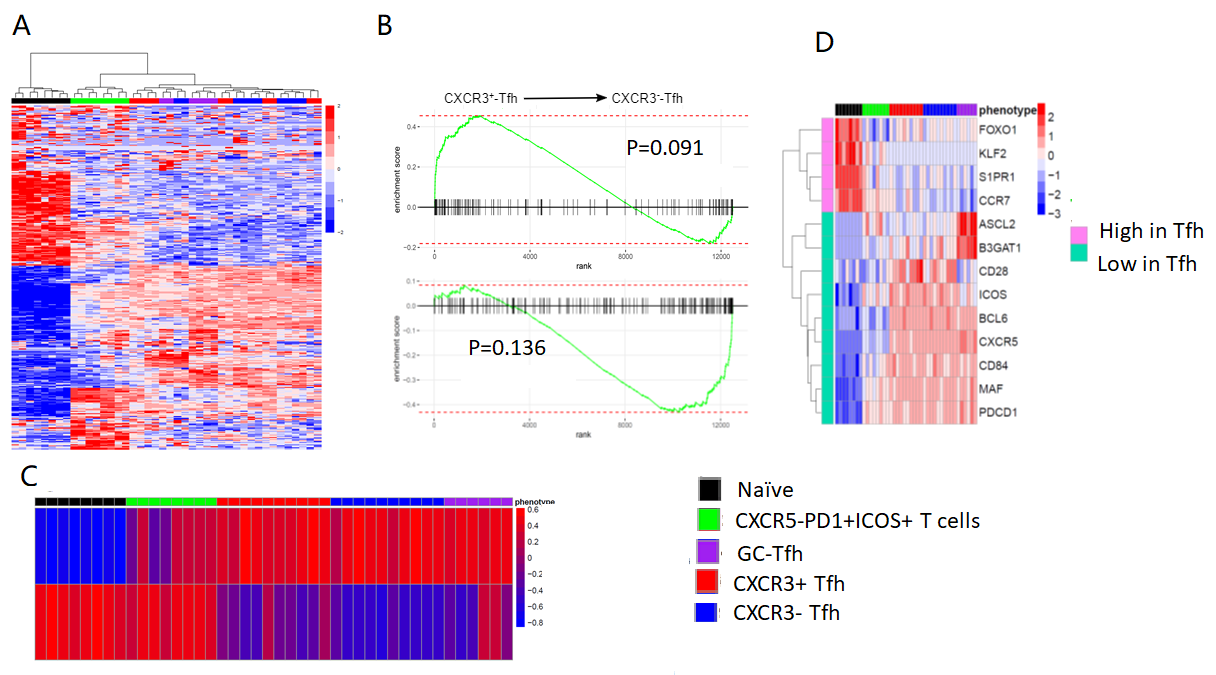


**Figure S15. CXCR3^+^T_FH_ expresses similar T_FH_ marker genes with CXCR3^-^T_FH_ population.** (A). Heatmap visualization of Tonsil T_FH_ marker genes (see methods) expressing across all the LN samples; (B). GSEA enrichment analysis between CXCR3^+^T_FH_ versus CXCR3^-^T_FH_ on genes upregulated in tonsil T_FH_ (Upper panel, gene set 3) and on genes down-regulated in tonsil T_FH_ (Bottom panel, gene set 4). (C). Heatmap plot shows the GSVA enrichment score of LN samples on gene set 3 (upper row) and gene set 4 (bottom row); (D). Heatmap visualization of curated T_FH_-related genes expressing across all the LN samples. Rows were labeled according to whether this gene should be up-regulated (pink) or down-regulated (green) in T_FH_ compared to non-T_FH_ in literature.

**Figure S16.** DEGs (Leukocyte migration, GO:0050900) between CXCR3^+^T_FH_ and CXCR3^-^T_FH_

**Figure S17. BHA (Bhattacharyya) similarity among all paired samples within LN.**


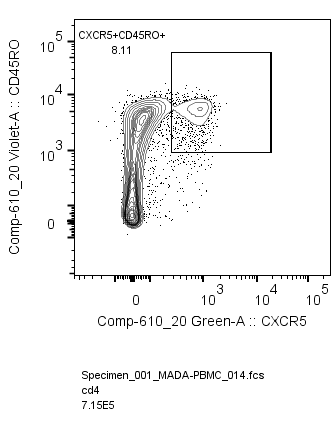

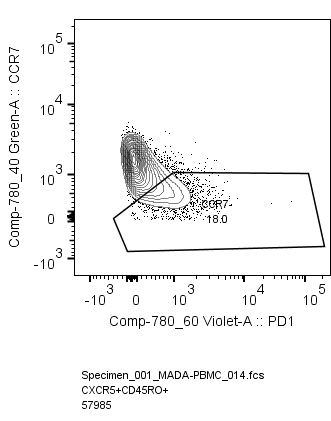


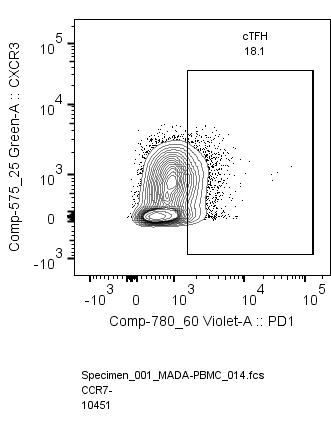


**Figure S18. Gating strategy for sorting circulating T_FH_ (cT_FH_).**


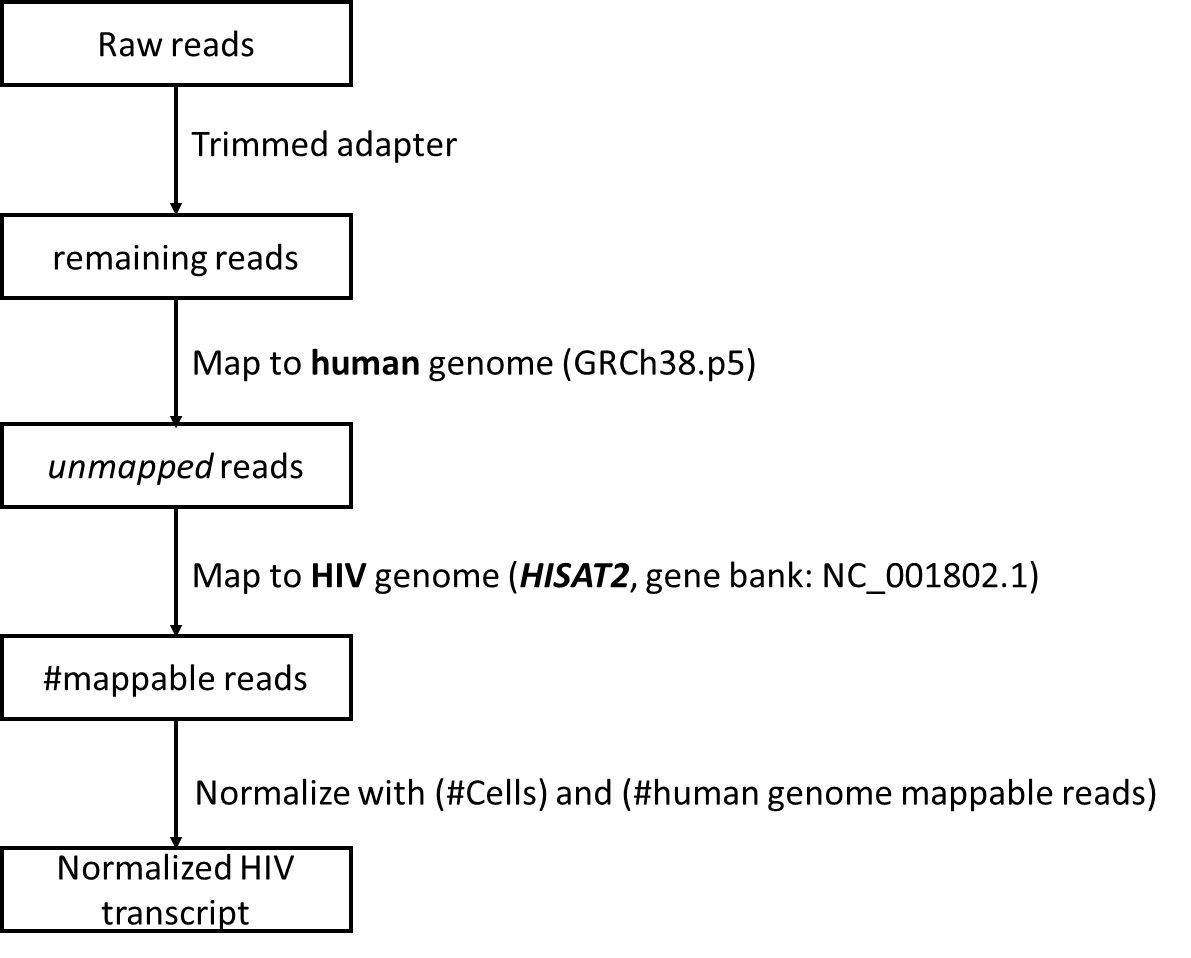


**Figure S19. Schematic of HIV transcript quantification from bulk RNA-seq data.**


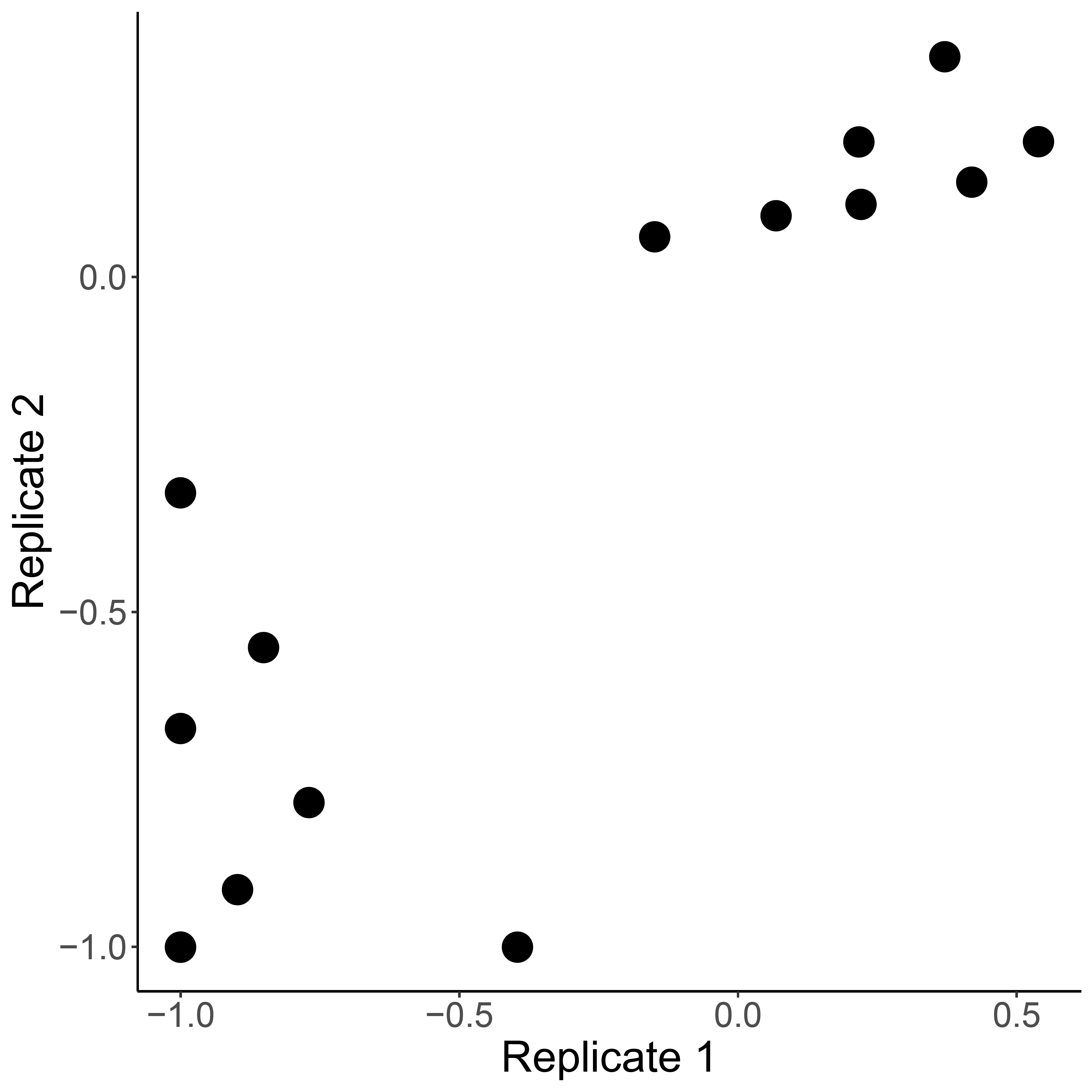


**Figure S20. Correlation between normalized number of HIV transcripts within two replicates of measured samples.** X axis is replicate 1 while Y axis is replicate 2.

**Figure S21. HIV transcript comparison between HIV^+^ and ART treated donors.** Histogram plot shows the normalized number of HIV transcript within each T cell population. One ART treated donor was used as a negative control.

**Table S1. Characteristics of donors in CyTOF analysis**


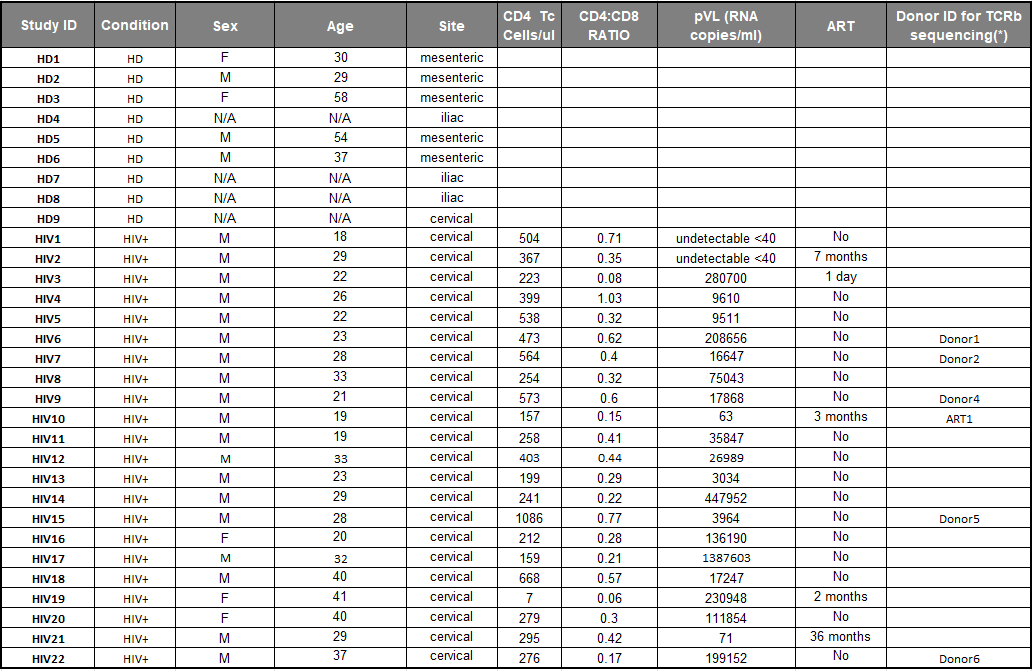


(*). The Donor ID in Table S5

**Table S2. Annotation and rational of clusters in CyTOF analysis**


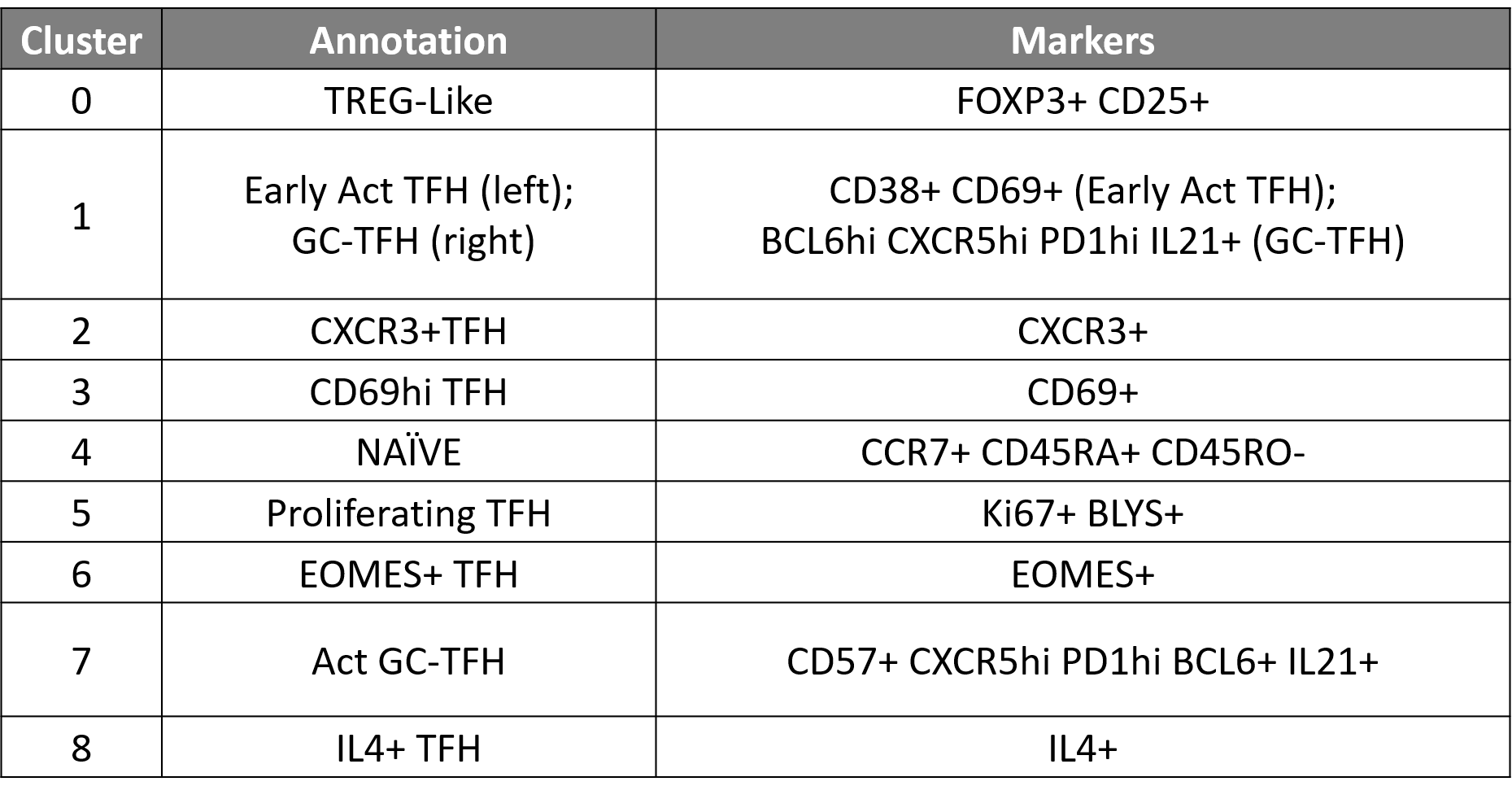


**Table S3. P-values of CyTOF populations compared on UMAP_1**


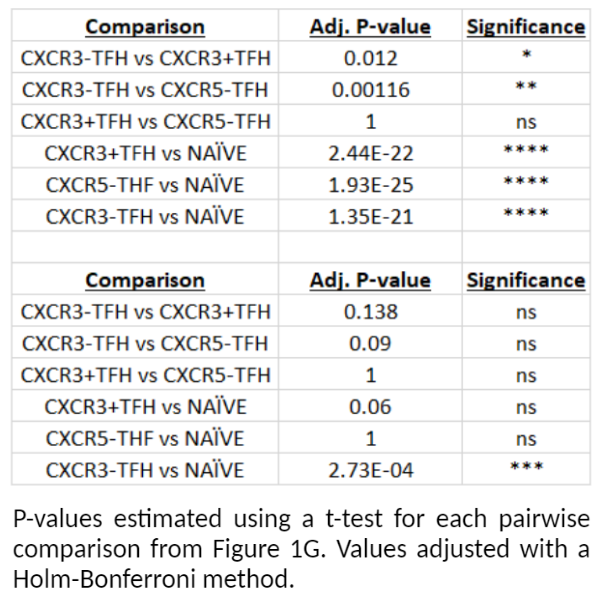


**Table S4. Primers used for TCRβ library amplification**

| **RT (TRBC-specific primer with 12-nucleotide molecular identifier):** | |
| --- | --- |
| huTRBC_12N | ACACTCTTTCCCTACACGACGCTCTTCCGATCT NNNNNNNNNNNN GACCTCGGGTGGGAACAC (N indicates random molecular barcode) |
| **1st PCR (partial Illumina adapters & TRBV-specific primers):** | |
| 1st PCR forward | ACACTCTTTCCCTACACGAC |
| 1st PCR reverse (TRBV-specific): | |
| TRBV1 | GACGTGTGCTCTTCCGATCTCTGACAGCTCTCGCTTATACCTTCA |
| TRBV2 | GACGTGTGCTCTTCCGATCTGCCTGATGGATCAAATTTCACTCTG |
| TRBV3 | GACGTGTGCTCTTCCGATCTAATGAAACAGTTCCAAATCGMTTCT |
| TRBV4 | GACGTGTGCTCTTCCGATCTCCAAGTCGCTTCTCACCTGAAT |
| TRBV5-1 | GACGTGTGCTCTTCCGATCTCGCCAGTTCTCTAACTCTCGCTCT |
| TRBV5-2 | GACGTGTGCTCTTCCGATCTTTACTGAGTCAAACACGGAGCTAGG |
| TRBV5-3 | GACGTGTGCTCTTCCGATCTCTCTGAGATGAATGTGAGTGCCTTG |
| TRBV5-4/5/6/7/8 | GACGTGTGCTCTTCCGATCTCTGAGCTGAATGTGAACGCCTTG |
| TRBV6-1 | GACGTGTGCTCTTCCGATCTTCTCCAGATTAAACAAACGGGAGTT |
| TRBV6-2/3 | GACGTGTGCTCTTCCGATCTCTGATGGCTACAATGTCTCCAGATT |
| TRBV6-4 | GACGTGTGCTCTTCCGATCTAGTGTCTCCAGAGCAAACACAGATG |
| TRBV6-5/6/7 | GACGTGTGCTCTTCCGATCTGTCTCCAGATCAAMCACAGAGGATT |
| TRBV6-8/9 | GACGTGTGCTCTTCCGATCTAAACACAGAGGATTTCCCRCTCAG |
| TRBV7-1 | GACGTGTGCTCTTCCGATCTGTCTGAGGGATCCATCTCCACTC |
| TRBV7-2 | GACGTGTGCTCTTCCGATCTTCGCTTCTCTGCAGAGAGGACTGG |
| TRBV7-3 | GACGTGTGCTCTTCCGATCTCTGAGGGATCCGTCTCTACTCTGAA |
| TRBV7-4/8 | GACGTGTGCTCTTCCGATCTCTGAGRGATCCGTCTCCACTCTG |
| TRBV7-5 | GACGTGTGCTCTTCCGATCTGGTCTGAGGATCTTTCTCCACCT |
| TRBV7-6/7 | GACGTGTGCTCTTCCGATCTGAGGGATCCATCTCCACTCTGAC |
| TRBV7-9 | GACGTGTGCTCTTCCGATCTCTGCAGAGAGGCCTAAGGGATCT |
| TRBV8-1 | GACGTGTGCTCTTCCGATCTAAGCTCAAGCATTTTCCCTCAAC |
| TRBV8-2 | GACGTGTGCTCTTCCGATCTATGTCACAGAGGGGTACTGTGTTTC |
| TRBV9 | GACGTGTGCTCTTCCGATCTACAGTTCCCTGACTTGCACTCTG |
| TRBV10-1/3 | GACGTGTGCTCTTCCGATCTACAAAGGAGAAGTCTCAGATGGCTA |
| TRBV10-2 | GACGTGTGCTCTTCCGATCTTGTCTCCAGATCCAAGACAGAGAA |
| TRBV11 | GACGTGTGCTCTTCCGATCTCTGCAGAGAGGCTCAAAGGAGTAG |
| TRBV12-1/2 | GACGTGTGCTCTTCCGATCTATCATTCTCYACTCTGAGGATCCAR |
| TRVB12-3/4/5 | GACGTGTGCTCTTCCGATCTACTCTGARGATCCAGCCCTCAGAAC |
| TRBV13 | GACGTGTGCTCTTCCGATCTCAGCTCAACAGTTCAGTGACTATCAT |
| TRBV14 | GACGTGTGCTCTTCCGATCTGAAAGGACTGGAGGGACGTATTCTA |
| TRBV15 | GACGTGTGCTCTTCCGATCTGCCGAACACTTCTTTCTGCTTTCT |
| TRBV16 | GACGTGTGCTCTTCCGATCTATTTTCAGCTAAGTGCCTCCCAAAT |
| TRBV17 | GACGTGTGCTCTTCCGATCTCACAGCTGAAAGACCTAACGGAAC |
| TRBV18 | GACGTGTGCTCTTCCGATCTATTTTCTGCTGAATTTCCCAAAGAG |
| TRBV19 | GACGTGTGCTCTTCCGATCTGTCTCTCGGGAGAAGAAGGAATC |
| TRBV20-1 | GACGTGTGCTCTTCCGATCTGACAAGTTTCTCATCAACCATGCAA |
| TRBV21-1 | GACGTGTGCTCTTCCGATCTCAATGCTCCAAAAACTCATCCTGT |
| TRBV22-1 | GACGTGTGCTCTTCCGATCTAGGAGAAGGGGCTATTTCTTCTCAG |
| TRBV23-1 | GACGTGTGCTCTTCCGATCTATTCTCATCTCAATGCCCCAAGAAC |
| TRBV24-1 | GACGTGTGCTCTTCCGATCTGACAGGCACAGGCTAAATTCTCC |
| TRBV25-1 | GACGTGTGCTCTTCCGATCTAGTCTCCAGAATAAGGACGGAGCAT |
| TRBV26 | GACGTGTGCTCTTCCGATCTCTCTGAGGGGTATCATGTTTCTTGA |
| TRBV27 | GACGTGTGCTCTTCCGATCTCAAAGTCTCTCGAAAAGAGAAGAGGA |
| TRBV28 | GACGTGTGCTCTTCCGATCTAAGAAGGAGCGCTTCTCCCTGATT |
| TRBV29-1 | GACGTGTGCTCTTCCGATCTCGCCCAAACCTAACATTCTCAA |
| TRBV30 | GACGTGTGCTCTTCCGATCTCCAGAATCTCTCAGCCTCCAGAC |
| **2nd PCR (Illumina index primers):** | |
| 2nd PCR forward | AATGATACGGCGACCACCGAGATCTACAC XXXXXXXX ACACTCTTTCCCTACACGAC (X indicates fixed library index) |
| 2nd PCR reverse | CAAGCAGAAGACGGCATACGAGATAA XXXXXX GTGACTGGAGTTCAGACGTGTGCTCTTCCGATCT (X indicates fixed library index) |

**Table S5. Summary of Donor and TCRβ sequencing information**

| **DONOR ID** | **Phenotype** | **Number of cells** | **Number of TCRβ transcripts** |
| --- | --- | --- | --- |
| Donor1 | Naïve | 10000 | 16966 |
|  | CXCR5^-^PD1^+^ICOS^+^ | 2500 | 3376 |
|  | GC-Tfh | 10000 | 35574 |
|  | CXCR3^+^-Tfh | 5000 | 4605 |
|  | CXCR3^-^-Tfh | 7500 | 7003 |
| Donor2 | Naïve | 10000 | 23261 |
|  | CXCR5^-^PD1^+^ICOS^+^ | 2500 | 4571 |
|  | GC-Tfh | 10995 | 51414 |
|  | CXCR3^+^-Tfh | 5000 | 5924 |
|  | CXCR3^-^-Tfh | 5000 | 5190 |
| Donor3 | Naïve | 10000 | 18014 |
|  | CXCR5^-^PD1^+^ICOS^+^ | 7500 | 8152 |
|  | GC-Tfh | 10000 | 42944 |
|  | CXCR3^+^-Tfh | 10000 | 9862 |
|  | CXCR3^-^-Tfh | 10000 | 10320 |
| Donor4 | Naïve | 10000 | 12891 |
|  | CXCR5^-^PD1^+^ICOS^+^ | 2500 | 2567 |
|  | GC-Tfh | 10227 | 30886 |
|  | CXCR3^+^-Tfh | 5000 | 3744 |
|  | CXCR3^-^-Tfh | 5000 | 5330 |
| Donor5 | Naïve | 10000 | 5850 |
|  | CXCR5^-^PD1^+^ICOS^+^ | 2515 | 3277 |
|  | GC-Tfh | 10000 | 11217 |
|  | CXCR3^+^-Tfh | 5000 | 6676 |
|  | CXCR3^-^-Tfh | 2500 | 5582 |
| Donor6 | Naïve | 10000 | 4611 |
|  | CXCR5^-^PD1^+^ICOS^+^ | 4085 | 3611 |
|  | GC-Tfh | 1464 | 2526 |
|  | CXCR3^+^-Tfh | 2249 | 2566 |
|  | CXCR3^-^-Tfh | 2476 | 3320 |
| Donor7 | Naïve | 10000 | 6186 |
|  | CXCR5^-^PD1^+^ICOS^+^ | 10000 | 12336 |
|  | GC-Tfh | 10000 | 12306 |
|  | CXCR3^+^-Tfh | 10000 | 10068 |
|  | CXCR3^-^-Tfh | 10000 | 10625 |
| ART1 | Naïve | 10000 | 7473 |
|  | CXCR5^-^PD1^+^ICOS^+^ | 2500 | 2854 |
|  | GC-Tfh | 10000 | 8930 |
|  | CXCR3^+^-Tfh | 5000 | 15126 |
|  | CXCR3^-^-Tfh | 5000 | 6755 |

**Table S6. Primers for bulk RNA-seq**

SSII_TSO = AAGCAGTGGTATCAACGCAGAGTACATrGrG+G

RT_dT30VN = AAGCAGTGGTATCAACGCAGAGTACTTTTTTTTTTTTTTTTTTTTTTTTTTTTTTVN

RT_TSPCR = AAGCAGTGGTATCAACGCAGAGT

Nextera_rev = CAAGCAGAAGACGGCATACGAGAT[i7]GTCTCGTGGGCTCGG

Nextera_for = AATGATACGGCGACCACCGAGATCTACAC[i5]TCGTCGGCAGCGTC

**Table S7. Genes contributed to PC2 in the PCA analysis. Top 100 genes most positively or negatively correlated to PC2 were included**


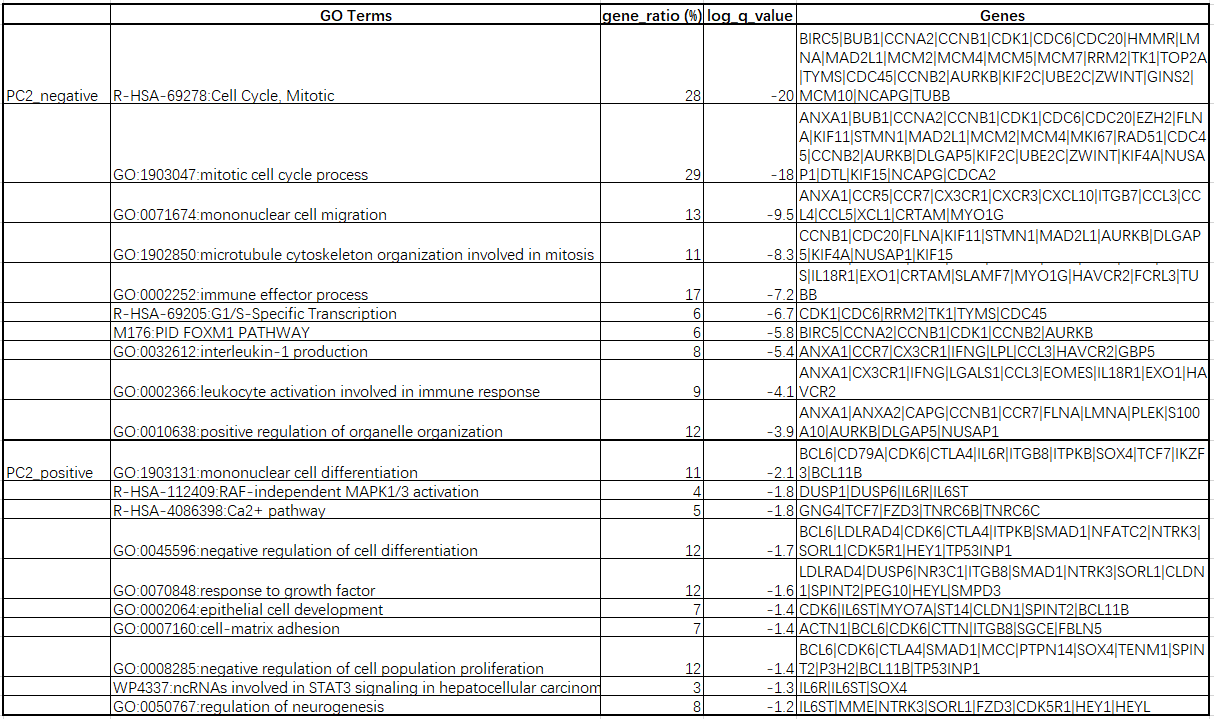


**Table S8. DEGs between CXCR3+/-Tfh cells**

| **gene.name** | **baseMean** | **log2FoldChange** | **lfcSE** | **stat** | **pvalue** | **padj** |
| --- | --- | --- | --- | --- | --- | --- |
|  |  |  |  |  |  |  |
| GSTA4 | 3.807621012 | -5.838022427 | 1.68275726 | -3.469319412 | 0.000521779 | 0.035723309 |
| ZNF683 | 3.209856864 | -5.585600326 | 1.658526944 | -3.367808009 | 0.000757683 | 0.045521524 |
| DIRAS2 | 13.56765149 | -5.312844867 | 1.221287656 | -4.350199433 | 1.36E-05 | 0.002736557 |
| RNASE6 | 4.990368393 | -5.027193133 | 1.394793881 | -3.604255226 | 0.000313049 | 0.025273365 |
| NELL2 | 21.98486776 | -3.926649407 | 1.010953998 | -3.884102951 | 0.000102708 | 0.011805811 |
| HIST1H2AH | 7.020255113 | -3.804976799 | 0.714706819 | -5.323828876 | 1.02E-07 | 5.09E-05 |
| HIST1H1E | 97.91344069 | -3.184305658 | 0.371701974 | -8.566824725 | 1.06E-17 | 6.66E-14 |
| CXCR3 | 557.6990335 | -2.917949443 | 0.463867489 | -6.290480605 | 3.16E-10 | 3.97E-07 |
| HIST1H1D | 55.0900653 | -2.555047879 | 0.282020271 | -9.059802213 | 1.31E-19 | 1.64E-15 |
| FCRL3 | 122.6766659 | -2.27352903 | 0.525520283 | -4.326244113 | 1.52E-05 | 0.002879265 |
| C16orf59 | 17.10849705 | -2.227047653 | 0.654821251 | -3.401000883 | 0.000671396 | 0.042558455 |
| ZNF407 | 171.0650398 | -2.072022056 | 0.366628381 | -5.651559358 | 1.59E-08 | 1.33E-05 |
| GLIPR2 | 71.33666407 | -2.005696409 | 0.428093031 | -4.685188182 | 2.80E-06 | 0.000796454 |
| IFNG | 185.4497357 | -1.967684504 | 0.513079848 | -3.835045389 | 0.000125541 | 0.013677431 |
| CASC5 | 88.60615191 | -1.953040953 | 0.564678636 | -3.458676897 | 0.000542836 | 0.03636999 |
| PKMYT1 | 63.48439068 | -1.940540367 | 0.307505904 | -6.310579218 | 2.78E-10 | 3.87E-07 |
| RNF157 | 7.743029338 | -1.922750305 | 0.555022051 | -3.464277326 | 0.000531658 | 0.036170413 |
| GPR171 | 95.63722431 | -1.917596297 | 0.543289517 | -3.529602977 | 0.000416184 | 0.031411841 |
| HIST1H1B | 12.78780486 | -1.87887328 | 0.534386483 | -3.515944619 | 0.000438192 | 0.032288909 |
| TROAP | 39.83314069 | -1.808975626 | 0.511246975 | -3.538359569 | 0.000402621 | 0.030572383 |
| F2RL2 | 92.18500412 | -1.800479071 | 0.51237985 | -3.513953705 | 0.00044149 | 0.032288909 |
| HIST1H2AM | 16.2129005 | -1.719733351 | 0.389365306 | -4.416760618 | 1.00E-05 | 0.00220227 |
| ATAD5 | 131.0350673 | -1.696591871 | 0.329059612 | -5.15587999 | 2.52E-07 | 0.000102475 |
| ARHGAP11A | 121.4736431 | -1.674791265 | 0.348647955 | -4.803674426 | 1.56E-06 | 0.000500453 |
| TAF15 | 70.44941478 | -1.608008855 | 0.427437217 | -3.761976707 | 0.000168576 | 0.017171427 |
| MB21D1 | 21.55604824 | -1.583126648 | 0.475224308 | -3.331325064 | 0.000864336 | 0.049242683 |
| CXCR6 | 152.4653135 | -1.578716342 | 0.44589698 | -3.540540556 | 0.000399308 | 0.030572383 |
| IL10 | 302.2185182 | -1.527587053 | 0.411076916 | -3.71606138 | 0.000202352 | 0.018919954 |
| CST7 | 246.2252238 | -1.490720889 | 0.426973161 | -3.491369069 | 0.000480552 | 0.033824917 |
| GBP1 | 431.872959 | -1.444392711 | 0.305187266 | -4.732807926 | 2.21E-06 | 0.000660562 |
| ITGB7 | 136.3192973 | -1.408114678 | 0.342048106 | -4.116715326 | 3.84E-05 | 0.005664733 |
| FAM111B | 111.2548243 | -1.404601971 | 0.401338236 | -3.499796044 | 0.000465614 | 0.033526903 |
| RINL | 81.19042729 | -1.371884071 | 0.271836234 | -5.046729977 | 4.49E-07 | 0.000170636 |
| U2AF2 | 41.41299605 | -1.284382406 | 0.320061507 | -4.012923694 | 6.00E-05 | 0.007667148 |
| MCM2 | 395.9235239 | -1.277937804 | 0.23306648 | -5.483147131 | 4.18E-08 | 3.08E-05 |
| PRR11 | 74.23051018 | -1.257073104 | 0.346109409 | -3.632010778 | 0.000281221 | 0.023741842 |
| MKI67 | 635.2331938 | -1.216676987 | 0.324077463 | -3.7542783 | 0.000173842 | 0.017424495 |
| ASPM | 188.836112 | -1.213386939 | 0.30020742 | -4.041828608 | 5.30E-05 | 0.007145032 |
| UHRF1 | 171.4737121 | -1.210104198 | 0.351216373 | -3.445466362 | 0.000570075 | 0.037395124 |
| CCL5 | 135.4769339 | -1.191832982 | 0.24164662 | -4.932131808 | 8.13E-07 | 0.000275425 |
| ABCC1 | 178.7529858 | -1.157178326 | 0.24858588 | -4.655044469 | 3.24E-06 | 0.00090184 |
| ZWINT | 313.1364811 | -1.134456691 | 0.264862959 | -4.283183638 | 1.84E-05 | 0.003251152 |
| GPRIN3 | 706.4726943 | -1.131346006 | 0.158240335 | -7.149542542 | 8.71E-13 | 1.82E-09 |
| NOD2 | 110.7566779 | -1.126892575 | 0.314433615 | -3.583880734 | 0.000338527 | 0.026844311 |
| PDE4B | 424.1812637 | -1.092217291 | 0.271543223 | -4.022259437 | 5.76E-05 | 0.007445392 |
| IRF8 | 460.9614911 | -1.077644922 | 0.161254654 | -6.682876386 | 2.34E-11 | 4.19E-08 |
| ACOT7 | 220.405339 | -1.06441753 | 0.258073309 | -4.124477405 | 3.72E-05 | 0.005542255 |
| ETV7 | 558.3302779 | -1.056329622 | 0.167375445 | -6.311138556 | 2.77E-10 | 3.87E-07 |
| CENPF | 399.7750613 | -1.043729135 | 0.27933259 | -3.736510426 | 0.000186592 | 0.018359417 |
| SAMHD1 | 292.3969328 | -1.021551878 | 0.236724284 | -4.315365796 | 1.59E-05 | 0.002979636 |
| PON2 | 437.6886816 | 1.014601934 | 0.255562653 | 3.970071219 | 7.19E-05 | 0.00874003 |
| PVALB | 186.1289938 | 1.065465968 | 0.170350623 | 6.254546935 | 3.99E-10 | 4.54E-07 |
| ID3 | 82.75347154 | 1.147996593 | 0.26124856 | 4.394269547 | 1.11E-05 | 0.002400942 |
| CXXC5 | 134.1071375 | 1.15448716 | 0.271022142 | 4.259752175 | 2.05E-05 | 0.003448416 |
| NRIP2 | 123.880043 | 1.182458091 | 0.281056665 | 4.207187509 | 2.59E-05 | 0.004153341 |
| ZNF747 | 36.88281042 | 1.200669352 | 0.306971477 | 3.911338488 | 9.18E-05 | 0.010848935 |
| AK9 | 65.56374386 | 1.249073096 | 0.368707509 | 3.387707233 | 0.000704795 | 0.042696149 |
| PTPN14 | 595.3244026 | 1.321655396 | 0.293698419 | 4.500042597 | 6.79E-06 | 0.001667108 |
| SLC7A10 | 172.7962858 | 1.356078659 | 0.367139616 | 3.693632064 | 0.000221074 | 0.020366407 |
| TSR3 | 82.19331486 | 1.359458433 | 0.348551191 | 3.900312113 | 9.61E-05 | 0.011249023 |
| SORL1 | 1103.404625 | 1.399091457 | 0.241181327 | 5.800994095 | 6.59E-09 | 5.90E-06 |
| DUSP1 | 9364.69529 | 1.456871295 | 0.377732197 | 3.856889365 | 0.000114839 | 0.012732913 |
| CD79A | 251.0640942 | 1.462653222 | 0.378347704 | 3.86589692 | 0.000110682 | 0.01249307 |
| BLOC1S4 | 64.40163103 | 1.489742514 | 0.339660142 | 4.385979775 | 1.15E-05 | 0.002451965 |
| CNIH3 | 234.190726 | 1.543778709 | 0.195561127 | 7.894098046 | 2.92E-15 | 9.16E-12 |
| TTC28 | 64.72130489 | 1.586039587 | 0.446262746 | 3.554048821 | 0.000379349 | 0.029705381 |
| LRP3 | 11.93374425 | 1.896184373 | 0.549517788 | 3.450633293 | 0.000559273 | 0.036879634 |
| IFNGR2 | 108.1085041 | 1.910259504 | 0.342302829 | 5.580612671 | 2.40E-08 | 1.88E-05 |
| ST14 | 100.4940395 | 1.923594154 | 0.389749847 | 4.935458388 | 8.00E-07 | 0.000275425 |
| SH3RF3 | 38.1280258 | 1.92949795 | 0.573600785 | 3.363834221 | 0.000768677 | 0.045521524 |
| ANTXR2 | 104.1746927 | 2.057768617 | 0.547809623 | 3.756357193 | 0.000172405 | 0.017419814 |
| SMPD3 | 133.9863339 | 2.085875961 | 0.391153095 | 5.332633154 | 9.68E-08 | 5.05E-05 |
| CA8 | 74.4350159 | 2.150166962 | 0.63947383 | 3.362400243 | 0.00077268 | 0.045521524 |
| CPXM1 | 65.14725681 | 2.180723339 | 0.538277067 | 4.051302708 | 5.09E-05 | 0.007012559 |
| GNAQ | 27.72173417 | 2.188643695 | 0.586867329 | 3.729367078 | 0.000191961 | 0.018359417 |
| HEY1 | 52.26480957 | 2.67235098 | 0.585378944 | 4.565164169 | 4.99E-06 | 0.001330483 |
| TEAD1 | 37.98128648 | 2.674116363 | 0.612847578 | 4.363428133 | 1.28E-05 | 0.002673689 |
| RAI2 | 32.65960461 | 2.852389676 | 0.552327982 | 5.164304121 | 2.41E-07 | 0.000102475 |
| EDN3 | 51.070462 | 2.920444882 | 0.71883228 | 4.062762572 | 4.85E-05 | 0.006826946 |
| RNF130 | 16.05764208 | 3.507697325 | 0.823823871 | 4.257824335 | 2.06E-05 | 0.003448416 |
| ZCCHC12 | 10.30816888 | 3.672365557 | 1.079466791 | 3.402018097 | 0.000668902 | 0.042558455 |
| PODXL2 | 5.995877141 | 3.727482437 | 0.82843727 | 4.499414223 | 6.81E-06 | 0.001667108 |
| F13A1 | 31.24796123 | 4.102653796 | 0.977239863 | 4.198205528 | 2.69E-05 | 0.004266813 |
| FAM3B | 15.75937589 | 4.175745434 | 0.803137354 | 5.199291772 | 2.00E-07 | 9.28E-05 |
| C20orf194 | 10.42086703 | 4.290457895 | 1.134970579 | 3.780237105 | 0.000156679 | 0.016223406 |
| SLC25A43 | 9.119938047 | 4.360844929 | 1.196707758 | 3.64403498 | 0.000268397 | 0.023045904 |
